# Role of extreme vertex design approach on the mechanical and morphological behaviour of residual soil composite

**DOI:** 10.1038/s41598-023-35204-6

**Published:** 2023-05-16

**Authors:** Imoh Christopher Attah, George Uwadiegwu Alaneme, Roland Kufre Etim, Christopher Brownson Afangideh, Kufre Primus Okon, Obeten Nicholas Otu

**Affiliations:** 1 Department of Civil Engineering, Akwa Ibom State University, Ikot Akpaden, Nigeria; 2 Department of Civil Engineering, Kampala International University, Kampala, Uganda; 3 Department of Civil Engineering, Akwa Ibom State Polytechnic, Ikot Osurua, Nigeria; 4 Department of Civil Engineering, University of Cross River State, Calabar, Nigeria

**Keywords:** Engineering, Materials science

## Abstract

This research work reports the usability of binary additive materials known as tile waste dust (TWD) and calcined kaolin (CK) in ameliorating the mechanical response of weak soil. The extreme vertex design (EVD) was adopted for the mixture experimental design and modelling of the mechanical properties of the soil-TWD-CK blend. In the course of this study, a total of fifteen (15) design mixture ingredients’ ratios for water, TWD, CK and soil were formulated. The key mechanical parameters considered in the study showed a considerable rate of improvement to the peak of 42%, 755 kN/m^2^ and 59% for California bearing ratio, unconfined compressive strength and resistance to loss in strength respectively. The development of EVD-model was achieved with the aid of the experimental derived results and fractions of component combinations through fits statistical evaluation, analysis of variance, diagnostic test, influence statistics and numerical optimization using desirability function to analyze the datasets. In a step further, the non-destructive test explored to assess the microstructural arrangement of the studied soil-additive materials displayed a substantial disparity compared to the corresponding original soil material and this is an indicator of soil improvement. From the geotechnical engineering perspective, this study elucidates the usability of waste residues as environmental friendly and sustainable materials in the field of soil re-engineering.

## Introduction

In the course of constructing civil engineering products such as highway infrastructures, buildings, embankments etc. necessitates the use of soil materials. Soil as a construction material forms a sensitive layer in road structures and this makes it valuable for both construction and other human activities. It may interest one to know that there are cases where the in situ soil contains a substantial quantity of montmorillonite clay mineral and it is termed as black cotton soil. Such soil possesses expansive tendencies, both montmorillonite and illite have high dominance in the structure of this soil and consequently accountable for swell-shrink behaviour associated with the soil. These clay minerals are pivotal menace to foundations of uncountable civil engineering products such as road embankments, roadways etc. However, during the wet period this soil material grasps water whereas during the dry period it drops it moisture. This seasonal alterations results to stability issues which will in return stress the foundation of civil engineering products like buildings and road pavements. Although a number of soil investigations have been documented on the numerous complications of using black cotton soil during construction activities^1,2^, it is almost impossible for civil engineers not to encounter these soil materials in the cause of construction, most especially in the Northern part of Nigeria. In the course of constructing infrastructural projects, the stable soils are utilised as infrastructural construction material whereas the unstable ones are replaced with soil materials of good geotechnical standing or improved via stabilization protocols by the incorporation of additives. The occurrence of these unstable soil materials has been a major setback for the construction industry most especially civil engineers, thus making the execution of infrastructural projects on unfavourable soil conditions practically unavoidable. The setbacks posed by the soil when encountered during infrastructural development are well reported in the literature^2,3^. To mitigate these setbacks, preventive options including soil replacement, chemical modification and special foundation systems are employed^4^. The mitigation option often applied in soft soil is amendment with conventional or nonconventional ameliorants. The act of amending soft soil via stabilization using conventional ameliorants is becoming an old-fashioned practice coupled with some environmental concerns. The production process of these ameliorant materials coupled with the discharge of greenhouse gases is one of the numerous threats posed by them. It is important to note that the amendment of soil with non-conventional ameliorants which are sustainable and economical, has proved to be very successful due to their ability to perform anticipated outcomes in the geotechnical parameters of soft clay soil compared to conventional ameliorants. Thus, the emerging advances in construction materials have proved that diverse ameliorants rich in alumina and silica such as sawdust ash—quarry dust^5^, cement kiln dust—rice husk ash^6^, cement kiln dust—metakaolin^7,8^, cement kiln dust^9^, periwinkle shell ash^10^, oyster shell ash^11,12^, quarry dust^13^, marble dust—rice husk ash^14^, coconut husk ash^15^, corncob ash^16^, locust bean waste ash^17,18^, groundnut shell ash^19–21^, lime-rice husk ash^22^, bambara nut shell ash^23^, yam peel ash^24^, metakaolin^25^, mine tailings^26^, palm bunch ash^27^, cement kiln dust-periwinkle shell ash^28,29^, cow bone ash—waste glass powder^30^, sawdust ash—lime^31^, rice husk ash—sisal fibre^32^, coffee husk ash^33^, construction waste^34^, sawdust^35^, nanosilica^36^, electric arc furnace dust^37^, iron ore tailing^38^ and so on have been utilised in the amendment studies of deficient soils and concrete structures. Conclusively, as a result of the positive outcomes recorded by the incorporation of solid wastes in soil amelioration studies, a comprehensive review was carried out on the trends in expansive soil stabilization using calcium-based stabilizer materials^39^. From their all-inclusive review study, it was documented that calcium-based stabilizer materials (CSMs) display pozzolanic tendencies thereby influencing the engineering, geotechnical and microstructural properties of clayey soil materials. However, it is worth noting that the main motivation behind the use of these ameliorants/additives is the decline in greenhouse gas production and the cost of purchasing conventional binding agents (Cement and lime).

Remarkable leaps and bounds have been accomplished in the field of civil engineering construction materials using single non-conventional ameliorants. Attah et al.,^25^ studied modelling and predicting CBR values of lateritic soil treated with metakaolin (non-conventional ameliorant) and concluded that there was an enhancement in the strength values of the soil when 20% metakaolin was added by air-dried weight of the lateritic soil. Interestingly, a different approach has been explored by soil experts, which involves the use of multi non-conventional additives thereby in some cases incorporating non-conventional ameliorants combined with a little fraction of the conventional binders for soil improvement. The essence of this recent approach is to ensure that there will be adequate pozzolana in the soil matrix reacting with silica and alumina which will activate high strength in the studied soil. Similarly, Ekpo et al.,^28^ also combined periwinkle shell ash and cement kiln dust to strengthen selected lateritic soils. In a related development, Etim et al.,^40^ improved the properties of lateritic soil by using the combination of lime and periwinkle shell ash and reported the microstructural and geotechnical behaviour of both the untreated and optimally treated soils. The enhancement in the soil properties was linked with the pozzolanic interplay as a result of the incorporation of lime-periwinkle shell ash blend into the soil.

It is worth mentioning, the recent experimental investigation carried out by soil experts has proved the continuous efforts towards the amelioration of deficient soil using single or multi-additives. In the course of ameliorating this deficient soil, considerable improvement in the properties of the soil has been documented. However, notwithstanding the improvement recorded by most soil experts, only a few have deployed optimization tools to arrive at an appropriate target in the properties of the soil. Thus, the latest innovations in Civil engineering practice have prompted specialists in materials and construction engineering to deploy optimization techniques with the sole aim of building precise and reliable models for elucidating engineering problems^9,41,42^. This development occasioned the possibility of formulating equations to become accustomed to the technical hitches connected with modelling the behaviour of soil materials^43^. Remarkably, numerous materials and construction engineering scientists have looked into the achievability of utilizing diverse optimization tools and the conducted experimental outcomes revealed optimistic outcomes such as: extreme vertex design (EVD)^44^, response surface methodology (RSM)^34,45^, artificial neural network (ANN)^46^, gene expression programming (GEP)^47,48^, Taguchi optimization method^49,50^, Scheffe’s optimization approach^7^, artificial intelligence models^51^ and so on. In view of this, the EVD approach is a mixture blending system which utilises a smaller fraction within the simplex and has been widely used in Civil engineering. Also, EVD is an offline optimization measure whose objective is to optimise most engineering parameters and processes. It also aids in the design of experiment process and achieving the best possible mixture combinations during optimization protocols. Moreover, to the best of the authors’ knowledge, the major advantage of EVD compared to other optimization strategies is the flexibility in terms of the imposition of other constraints factor levels by specifying both the upper and lower bounds on the components via the designation of linear constraints for blends^52^. This has prompted numerous research experts to explore the utilization of EVD in Civil engineering materials^9,49,52^.

Moving onward, recent investigation have shown the level of potency displayed by residues such CK or TWP when utilised as single additive or combined with other additives in geo-engineering protocols. Howbeit, to the best of the author’s knowledge there exist infinitesimal or no known studies documented in open literature that evaluates the role of blending CK-TWP in ameliorating the geotechnical performance of an expansive soil using the principles EVD. Hence, this research becomes necessary and crucial. In this manuscript, one of the author’s hotspots is the use of scanning electron microscopy (SEM) to elucidate mutual interference between the studied soil and additives/ameliorants thereby detecting the amelioration potentials of the additives/ameliorants. The main focus of employing SEM is to also visualise soil-ameliorants interplay at the microscale efficiently and as well provide both qualitative and quantitative reports. The motivation behind the use of SEM in this study is not unconnected with the scanty use of qualitative tests in optimization studies and as well close the gap of the existing studies in soil re-engineering. Finally, it is presumed that blending CK-TWP would be a cheaper and sustainable option compared to the conventional stabilization agents such as lime, bitumen and cement. The benefit of the present investigation will entail in two folds: enhancing the soil performance and as well re-utilising / disposing of waste. Moreover, it is believed that the upshot of this study will create an enabling strategy for waste management.

## Materials and methods

### Materials

**Figure 1:**
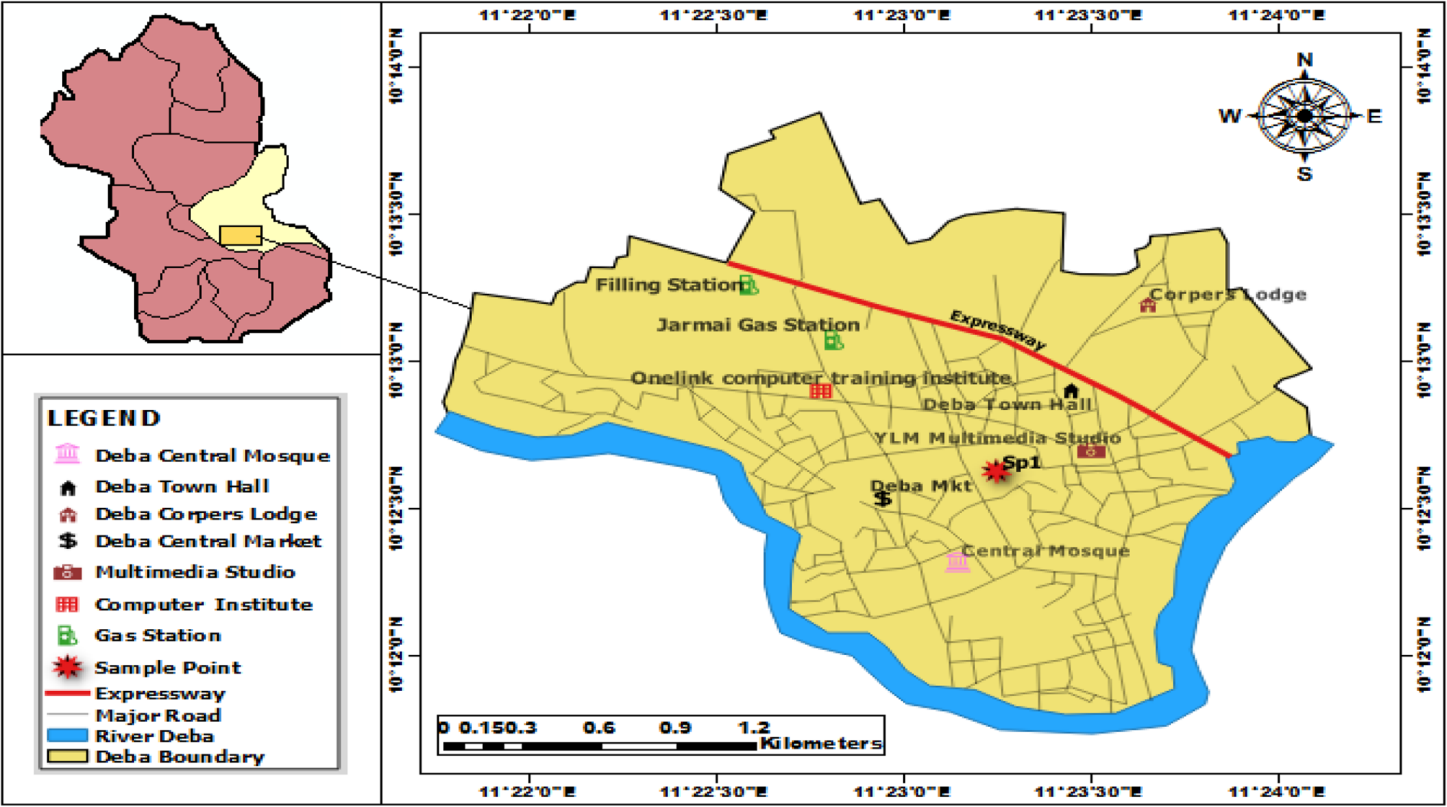
Location map of unaltered expansive soil material used in the paper generated using Arc GIS 10.1 software.

**Figure 2:**
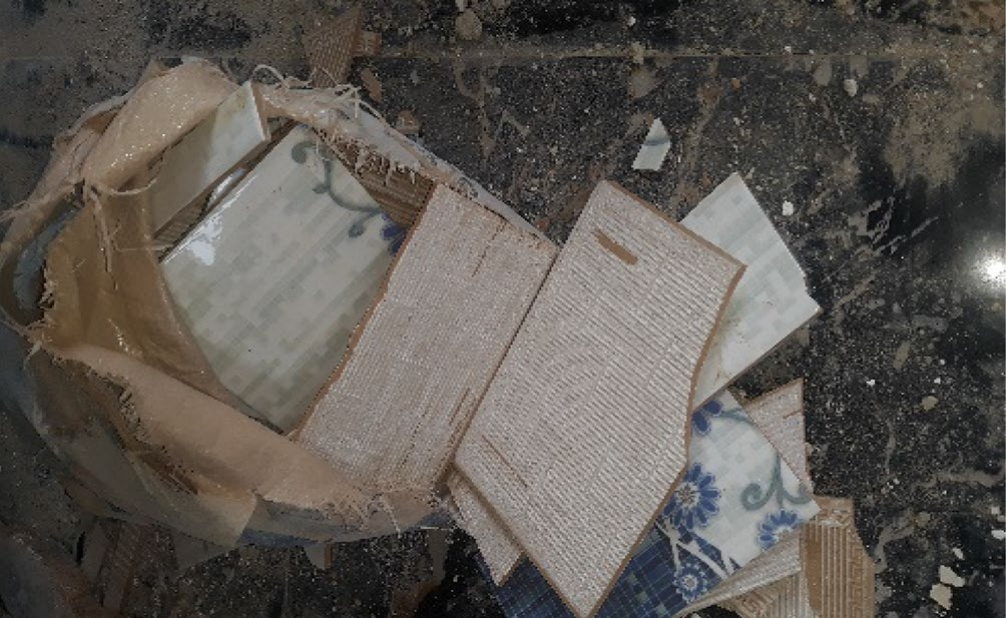
Broken tiles obtained from construction site in Uyo metropolis, Akwa Ibom Nigeria.

**Figure 3:**
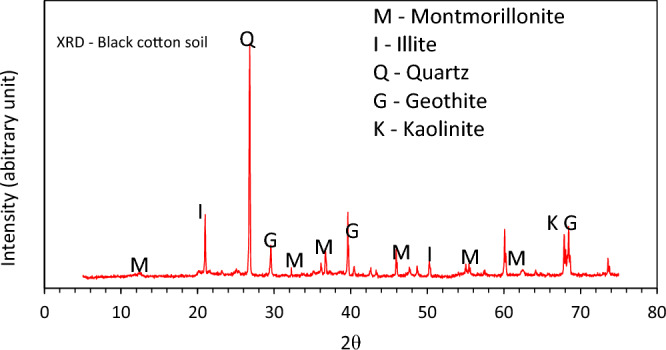
X-ray diffraction outcome of untreated ECS.

A potentially fine-grained soil also known as black cotton soil engaged in this current optimization exercise was acquired from its deposit in the municipality of Deba, Gombe State, Nigeria (which lies within latitude 10° 12ʹ 42.73ʹʹ N, longitude 11° 23ʹ 13.56ʹʹ E) as seen in the GIS plot in Fig. 1. The soil understudy is known to have a distinctive mineralogical and plasticity response. After excavating the soil material at an average depth of about 1.0 m, it was stacked and transported to the laboratory for the experimentation exercise. Before the experimental tests, it was sundried, pulverised and sieved with the relevant British Standard (BS) sieves. The two additive materials include: calcined kaolin (CK) and tile waste dust (TWD). The raw kaolin was gotten from a locality along the Enugu-Port Harcourt Highway. It was subjected to a controlled thermal activation/calcination and the ash residue gotten after the calcination protocols were allowed to cool and sieved via a 75 μm sieve to obtain a finely powdered substance for experimentation. The calcined kaolin had a specific gravity of 2.58. The tile wastes were gotten from a construction site in the Uyo metropolis, having a specific gravity of 2.75 as shown in Fig. 2. The dominant clay minerals obtainable in the unaltered soil were detected via means of the X-ray diffraction strategy whereas the elemental oxide compositions of the additive materials used in the study (soil, calcined kaolin and tile waste dust) were assessed utilizing the X-ray fluorescent strategy as shown in Fig. 3.

### Experimental techniques

British Standard approach was used to characterize the basic parameters of the unaltered soil which include: grain size distribution, Atterberg limits, specific gravity, natural moisture content, soaked California bearing ratio (CBR), 7 days unconfined compressive strength test (UCS) and resistance to loss in strength (RLS). In this study, the research methods involved experimental design using EVD, laboratory tests and optimization studies. They four number of constituent materials considered are as follows: CK, TWD, soil and water. The proportioning of the test materials were determined by a mix ratio using the EVD approach. The values served as the percentage by weight of the dry solid added in evaluating the considered responses for the study (CBR, UCS and RLS) as established by British Standards. However, in the course of this study it can be seen that studied soil in it natural form has poor geotechnical performance and as such it calls for soil improvement. Secondly, the utilisation of TWD and CK as additive materials was connected to some reasons such as: [i] Incorporation of both TWD and CK will ascertain that there will be enough pozzolana in the soil matrix to react with both silica and alumina components of the soil in the presence of moisture. [ii] Both TWD and CK are waste materials, reutilising them will curb environmental nuisance.

#### California bearing ratio test (CBR)

The procedure established by British Standard was engaged to find out the CBR values of virgin soil samples as well as the soil-additives admixed with the mix ratios developed by EVD strategy. The guiding principles of the British Standard Light compaction strategy were utilized in executing these experimentation exercises on the soil materials. This test involves the use of 2360 cm^3^ CBR mould and compacting the soil-additives uniformly with a 2.5 kg rammer in three layers. Thereafter, the compacted soil samples were subjected to a curing duration of 6 days. On completion of the 6 days of curing, the CBR samples were submerged in water for 48 h before testing (Nigerian General Specification)^53^.

#### Unconfined compressive strength test (UCS)

Expansive soil material was subjected to amelioration protocols with the mixtures of CK and TDW via the EVD method. Based on the outlines ascribed in BS 1377 part 4^54^, the soil mixtures were meticulously mixed and utilised for the UCS experimentation. The soil samples were compacted using British Standard Light (BSL) compaction energy and were allowed to cure for 7 days. Before the curing, the specimens were exposed to loadings axially in the UCS machine and for each experimentation, two samples were taken and the average values were documented.

#### Durability

Durability assessment could as well be referred to as the resistance to loss in strength when exposing soil material to an unfriendly environment such as water. Considering tropical regions like ours, the durability assessment was conducted according to the steps outlined in Ola^55^, which involves the ratio of UCS of the specimen wax-cured for 7 days and de-waxed top and bottom before being soaked for another 7 days to the UCS of the specimen cured for 14 days.

#### Morphological interaction

Both the unaltered and multiple-additive blended soil were morphologically and analytically visualized via scanning electron microscopy (SEM). The Phenom world electron microscopy testing equipment was engaged for conducting this test. The SEM analysis provides a series of micrographs which unravels the bonding, surface texture and geometry of the specimen^56^. Lately, the deployment of qualitative analysis such as SEM in optimization studies has been the hotspot for experts in the field of materials and construction engineering^57–61^.

### Mixture components constraints formulation

The formulation of mixture ingredients proportions and the required number of experimental runs were obtained with the aid of relevant literature works, empirical relationships, expert judgement and practical economic considerations. These design considerations are taken for the design and formulation of the constraints of the components which are imposed on the factor levels of lower and upper regions. However, the defined boundary limits and the sum-to-one constraint would modulate the generation of mixture components’ ratios using actual coding as shown in Table 1. The derived fractions of mixture components would provide a fundamental base upon which the EVD model development can be achieved to evaluate the properties of the soil-CK-TWD blended mixture.

**Table 1:** Design constraints.

Mixture Coding:	Actual	
Low	Constraint	High
0.100	A:Water	0.200
0.050	B:TWD	0.450
0.050	C:CK	0.450
0.350	D:Soil	0.500
	A + B + C + D	1.000

#### Design of simplex and factor space

**Figure 4:**
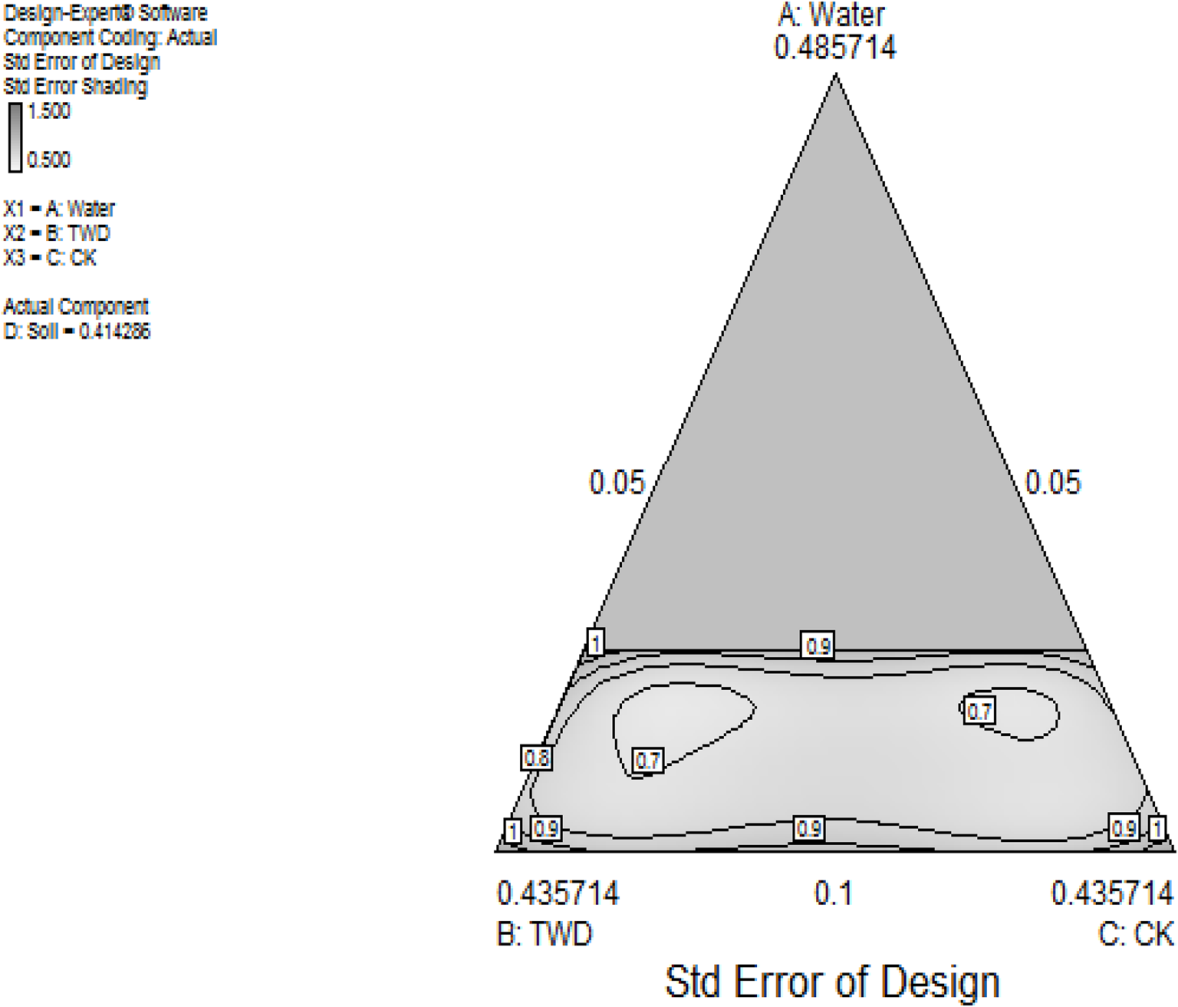
Contour plot of the constrained simplex.

**Figure 5:**
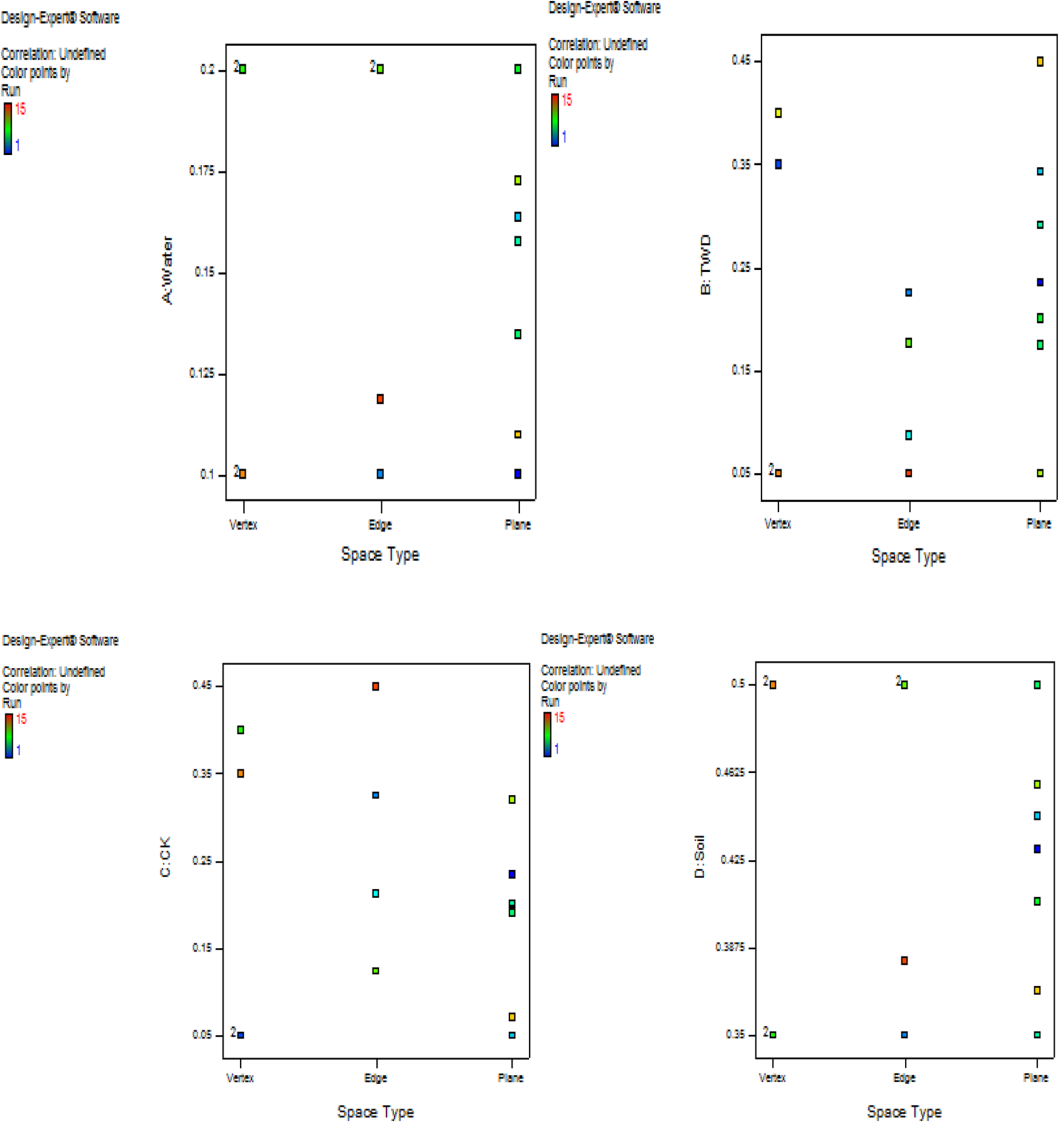
Experimental factor space of the components in a 4-component mixture space.

Moreover, as a result of multiple constraints imposition on the four mixture components, the resulting design factor space takes the form of a hyper-polyhedron simplex. Through the evaluation of the constraints of the components, the feasible region where the experimental region lies within the constrained portion of the simplex is derived. Also, through design matrix computation, the degrees-of-freedom (df) evaluation is achieved using a model of special-cubic as presented in Table 2. The essence is to guarantee the validity of the lack-of-fit test where fewer degrees of freedom would result in the inability of the test to identify lack-of-fit. The simplex factor space and contour plot for the 4-component mixtures were obtained with the help of design expert software and show the positioning of the experimental points within three space types of planes, vertices and edges of the simplex as shown in Figs. 4, 5. Information matrix measures which provide the leverage and build the type of the derived experimental points for the three space types were further calculated and presented in Table 3. The results obtained implied an average leverage value of 0.9333 with one lack of fit build type for experimental run 5 at edge space type.

**Table 2:** Design matrix Evaluation for mixture special cubic model using L_Pseudo.

Degrees of freedom for evaluation	
Model	13
Residuals	1
*Lack of fit*	*1*
*Pure error*	*0*
Corr total	14

**Table 3:** Measures derived from the information matrix.

Run	Leverage	Space type	Build type
1	0.9996	Plane	Model
2	0.9933	Vertex	Model
3	0.9999	Edge	Model
4	0.8871	Plane	Model
5	0.6481	Edge	Lack of fit
6	0.9994	Plane	Model
7	0.9951	Plane	Model
8	0.9950	Plane	Model
9	0.9970	Vertex	Model
10	0.7066	Edge	Model
11	0.7902	Plane	Model
12	0.9947	Vertex	Model
13	0.9987	Plane	Model
14	0.9968	Vertex	Model
15	0.9983	Edge	Model
Average =	0.9333		

Furthermore, the criterion optimality algorithm and I-optimal designs also known as IV (integrated variance) which offer the lowest average prediction of the variance across the experimental regions were carried out to evaluate the design experimental space to obtain a Condition Coefficient Matrix of 15,203.166; minimum, maximum and average mean variance of 0.23, 0.625 and 2.262 respectively; G-efficiency of 41.3% which is associated with the inverse of maximum variance; the scaled optimal criterion of 1803.097 and I-optimality value of 0.61945. The derived results implied adequate multicollinearity which would ensure acceptable model goodness of fit behaviour during model development.

#### Design of experimental mix proportions

Details obtained from the computed information matrix of the mixture design are utilized to formulate the components mixture ratio and the corresponding number of experimental runs. The actualization of these mixed ingredients fractions and the number of runs required were possible through the evaluation of the imposed components constraints and also from the criterion optimal algorithm computation outcome to ensure essential selection of the experimental points within the feasible factor space. Fifteen runs of experiments for the experimental exercise were obtained from the computation procedure as shown in Table 4; presenting the actual and pseudo components fractional values for the factor levels and providing the fundamental template for the evaluation of the 4-components mixture to stabilize the expansive soil using TWD-CK blends.

**Table 4:** Component proportion mixes for the experimental study.

Run	Actual values				Pseudo-coded values			
	A:Water	B:TWD	C:CK	D:Soil	A:Water	B:TWD	C:CK	D:Soil
1	0.100	0.235	0.235	0.430	0.000	0.412	0.411	0.177
2	0.100	0.350	0.050	0.500	0.000	0.667	0.000	0.333
3	0.100	0.225	0.325	0.350	0.000	0.389	0.611	0.000
4	0.164	0.343	0.050	0.444	0.141	0.650	0.000	0.208
5	0.200	0.088	0.212	0.500	0.222	0.084	0.361	0.333
6	0.157	0.291	0.201	0.350	0.128	0.536	0.336	0.000
7	0.135	0.174	0.191	0.500	0.077	0.276	0.313	0.333
8	0.200	0.200	0.193	0.407	0.222	0.333	0.317	0.127
9	0.200	0.050	0.400	0.350	0.222	0.000	0.778	0.000
10	0.200	0.176	0.124	0.500	0.222	0.280	0.164	0.333
11	0.173	0.050	0.320	0.457	0.162	0.000	0.601	0.238
12	0.200	0.400	0.050	0.350	0.222	0.778	0.000	0.000
13	0.110	0.450	0.071	0.369	0.022	0.889	0.046	0.043
14	0.100	0.050	0.350	0.500	0.000	0.000	0.667	0.333
15	0.119	0.050	0.450	0.382	0.041	0.000	0.889	0.070

## Results and discussion

As detailed in Table 5, is the elementary distinctive response of the representative expansive soil in its virgin state, while the particle size analysis curve is presented in Table 6. The particle size distribution exercise of the soil depicts a minute fraction of gravel (0.475%), a sand fraction of 27% and a large fraction of fine (72.028%). Sadly, the high fine content in the soil contributes to the expansive and plastic behaviour of the soil. Also, the strength indices of the studied soil were found not to meet the minimum benchmark. The oxide composition of constituent materials in this experimental investigation is shown in Table 7 which indicates good pozzolanic behaviour of the additives to improve the expansive soil’s mechanical characteristics for foundation purposes.

**Table 5:** Geochemical properties of untreated soil specimen.

Experiment	Standards	Quantity
Particle size (Gravel)	ASTM D 2487–11	0.475
Sand	ASTM D 2487–11	27
Silt	ASTM D 2487–11	62.028
Clay	ASTM D 2487–11	10
Natural moisture content, %	ASTM D 2216–10	20.10
LL, %	ASTM D 4318–10	56.30
PL, %	ASTM D 4318–10	27.60
PI, %	ASTM D 4318–10	28.70
AASHTO characterisation	AASHTO 1986	A-7–6-(14)
USCS	ASTM D 2487–11	CH
OMC, %	ASTM D 698–15	18
MDD, Mg/m^3^	ASTM D 698–15	1.61
Soaked CBR, %	ASTM D 1883–05	3
7 Days UCS, kN/m^2^	ASTM D 2166–16	102
Gs	ASTM D 854–15	2.40
Colour	–	Greyish black
Main clay mineral	–	Montmorillonite

**Table 6:** Particle size fraction of study soil.

D (mm)	Passing (%)
3.350	99.78
2.000	99.305
1.700	96.915
0.850	95.095
0.425	89.15
0.300	87.025
0.212	84.75
0.075	71.995
0.039	45.000
0.029	30.555
0.020	27.917
0.016	22.566
0.011	18.333
0.008	15.575
0.007	13.083
0.005	9.967

**Table 7:** Oxide composition of constituent materials in this paper.

Oxide composition		SiO_2_	CaO	SO_3_	MgO	TiO_2_	Fe_2_O_3_	Al_2_O_3_	Na_2_O	K_2_O	LOI
Mass fraction (%)	*BCS	7.95	45.75	-	0.11	0.54	5.10	1.98	-	-	37.25
	**CK	52.72	0.18	0.99	0.09	-	1.72	42.20	-	-	0.25
	TWD	69.85	3.05	3.90	0.95	4.75	2.49	10.98	0.75	0.98	-

^[*61^,^[**62^.

**Figure 6:**
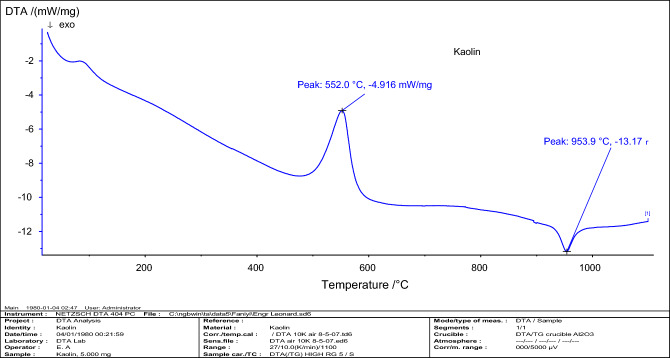
Differential scanning calorimetry (DSC) result of raw kaolin used in the paper.

Presented in Fig. 6 is the Differential scanning calorimetry (DSC) of the raw kaolin. The endothermic peak reactions took place between 552 and 953.9 °C this is connected with dihydroxylation of minerals^63^. This is a pointer towards the formation phase of calcined kaolin (CK). Interestingly, this DSC outcome is comparable to that documented by^64^ who highlighted that the formation phase of metakaolin sourced from Ajebo kaolin is about 550 °C.

### Impact of an optimal blend of ameliorants on California bearing ratio (CBR)

**Figure 7:**
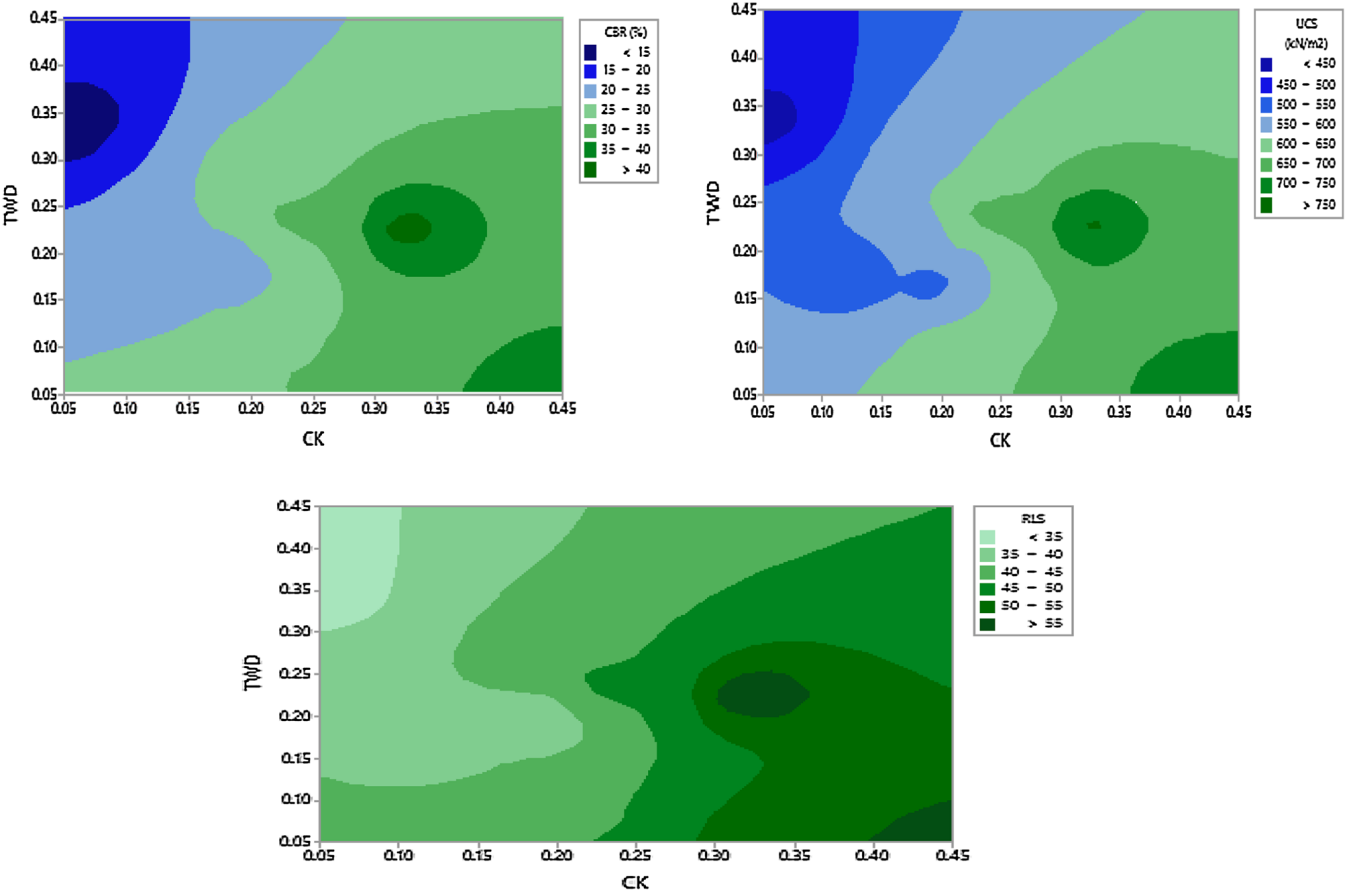
Contour plots showing effects of TWD-CK interaction on the mechanical strength response.

Table 8 presents the soaked CBR values for soil-calcined kaolin mixtures with tile waste dust. It shows that by adding the additives into the soil the CBR values of the treated soil mixtures yield prominent enhancement in terms of strength. The value of the CBR increased from an initial value of 9.41% to an optimal value of 61.4% as shown in a contour plot in Fig. 7. By comparing the CBR outcomes of the natural soil with the optimally treated soil sample, the rate of increment is about 552%. This remarking influence of an increase in CBR values results from some reasons. Primarily, the combination of calcium ions and water facilitates chemical interplay within the soil mixtures which thereby activates the establishment of new products such as CSH and CAH which has the affinity of influencing bonding properties. Another promising reason may be attributed to the high dominance of cementitious content in the soil matrix which would need hydration to ensure robust pozzolanic interplay as a result of the flocculation and agglomeration of clay particles that occurred. This chemical reaction forms a soil matrix that could efficiently enhance the non-bonded soil particles speedily and as well promote higher strength. A similar phenomenon was also documented by^9,13,59,60^.

**Table 8:** Test outcomes.

S/No	Actual ratios				Responses			Pseudo components				Mass conversion (kg)			
	Water	TWD	CK	Soil	CBR (S)	UCS (7 D)	RLS	X_1_	X_2_	X_3_	X_4_	Water	TWD	CK	Soil
1	0.10	0.24	0.24	0.43	32	675	47	0.00	0.41	0.41	0.18	1.62	3.81	3.81	6.96
2	0.10	0.35	0.05	0.50	11	450	33.5	0.00	0.67	0.00	0.33	1.62	5.67	0.81	8.1
3	0.10	0.23	0.32	0.35	42	755	59	0.00	0.39	0.61	0.00	1.62	3.65	5.26	5.67
4	0.16	0.34	0.05	0.44	11	410	33.5	0.14	0.65	0.00	0.21	2.65	5.55	0.81	7.19
5	0.20	0.09	0.21	0.50	30	640	44	0.22	0.08	0.36	0.33	3.24	1.42	3.44	8.1
6	0.16	0.29	0.20	0.35	27	595	43	0.13	0.54	0.34	0.00	2.55	4.72	3.26	5.67
7	0.13	0.17	0.19	0.50	23	520	39	0.08	0.28	0.31	0.33	2.18	2.82	3.09	8.1
8	0.20	0.20	0.19	0.41	24	580	37	0.22	0.33	0.32	0.13	3.24	3.24	3.12	6.6
9	0.20	0.05	0.40	0.35	36	735	55	0.22	0.00	0.78	0.00	3.24	0.81	6.48	5.67
10	0.20	0.18	0.12	0.50	21	515	36	0.22	0.28	0.16	0.33	3.24	2.85	2.01	8.1
11	0.17	0.05	0.32	0.46	33	670	51	0.16	0.00	0.60	0.24	2.8	0.81	5.19	7.4
12	0.20	0.40	0.05	0.35	17	480	33	0.22	0.78	0.00	0.00	3.24	6.48	0.81	5.67
13	0.11	0.45	0.07	0.37	19	480	34.5	0.02	0.89	0.05	0.04	1.78	7.29	1.15	5.98
14	0.10	0.05	0.35	0.50	35	705	54.5	0.00	0.00	0.67	0.33	1.62	0.81	5.67	8.1
15	0.12	0.05	0.45	0.38	39	730	59	0.04	0.00	0.89	0.07	1.92	0.81	7.29	6.18

### Impact of an optimal blend of ameliorants on unconfined compressive strength

The outcomes of unconfined compressive strength (UCS) carried out on the soil samples in the course of this experimental work are presented in Table 8. By comparing the UCS upshots of the unaltered soil to that of additive-altered soil, an amazing increase was witnessed with increasing calcined kaolin (CK) inclusion level irrespective of tile waste powder (TWP) content. The inclusion of these binary additives instigated an increase in UCS from 102 to 755 KN/m^2^ as shown in Table 3 and this translate to approximately seven times increment as shown in a contour plot in Fig. 7. The causes for this UCS improvement mechanism due to additive inclusion can be accredited to three reasons and they include: [1] Filling the voids within expansive soil grains by the binary additives during compaction which thereby imparts higher density to the soil, [2] alteration of the soil physical structure due to the higher specific gravity of the additives (i.e. CK and TWP having 2.58 and 2.5, respectively) in comparison with that of the BCS (2.40), and [3] formation of pozzolanic cementitious compounds between CK, TWP and BCS. In the presence of moisture, the soil reacts with the additives thereby releasing the calcium ions for replacement and exchange in the diffuse double layer of clay particles. The result aligns with that obtained by^9,62^.

### Impact of an optimal blend of ameliorants on resistance to loss in strength (RLS)

In the course of experimenting durability of the soil specimens, the obtained upshot is shown in Table 3. The durability value of 33% (which implies a loss in strength of 67%) was noticed to be the least possible value compared to 59% (which implies a loss in strength of 41%) being the utmost value. The least value of 33% and peak value of 59% were achievable as a result of mix combination ratios of 3.24:6.48:0.81:5.67 and 1.62:3.65:5.26:5.67 for water, tile waste dust, calcined kaolin and soil respectively. Consequently, the initial RLS value of 33% could be linked with the high dominance of swelling minerals in the parent soil whereas the peak value of 59% might not be unrelated with the presence of additives. It is important to note that the additives considered in this study are rich in the oxides of alumina and silica which are responsible for pozzolanic activities within the soil matrix and onward increment in mechanical strength. The peak RLS value in this study is low compared to the 80% requirement specified in^55^. Howbeit, considering the soaking duration of 7 days against the 4 days as earlier specified by Ola^55^, the peak RLS of 59% was recorded at mixture ratios of 1.62:3.65:5.26:5.67 for water, tile waste dust, calcined kaolin and soil respectively, looks promising. This experimental outcome is not far from the upshots of previous investigators who deployed other solid waste materials^7,65^.

### Impact of an optimal blend of ameliorants on micro-morphological test

#### Scanning electron microscope (SEM) observation

**Figure 8:**
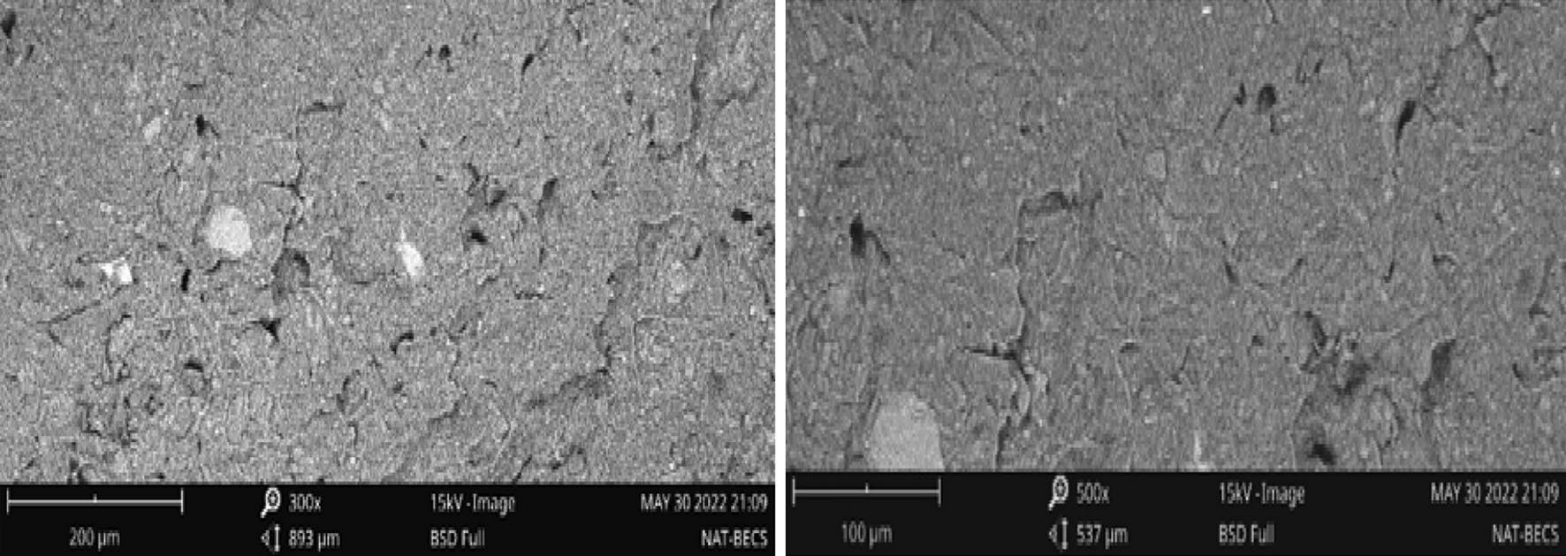
SEM micrographs of unstructured expansive soil at magnification levels of 300 × and 500x.

**Figure 9:**
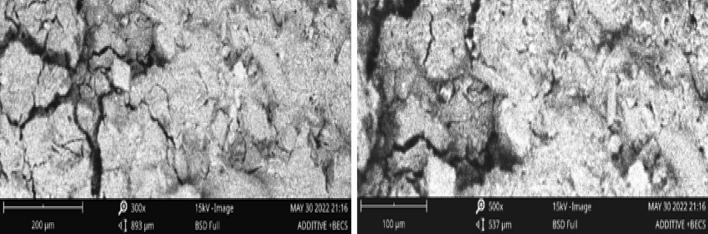
SEM micrographs of additive structured expansive soil at magnification levels of 300 × and 500x.

Lately, researchers with specialty in the field of materials and construction engineering have deployed a scanning electron microscope (SEM) to unravel the microstructural response of both soil and concrete materials^7,60,61,66^. The main motivation for the authors to subject both the unstructured and additive structured soil samples to the SEM testing was to establish the morphological amendment of the tested soil based on the optimization exercise. Figure 8 (a and b) shows the SEM micrograph of the raw soil sample at 300X and 500X magnifications. The unstructured soil material unveils a lumpy clay particle with a blackish appearance because of the absence of hydration products. As shown in the micrographs of the additive structured soil (Fig. 9), it results in a fairly whitish surface appearance with staggered bonding. On a second note, the inconsistencies noticed between the unstructured and additives-structured soil could be related to the incorporation of additives. The presence of these additives activated the dissolution of silicates thereby reacting with calcium and as well enhance the pozzolanic protocols within the soil matrix and the production of cemented materials. It is believed that the cemented composite bonds the aggregates and improves the strength response of the additive-structured soil^67–70^.

## Development, formulation and validation of the extreme vertex design (EVD) model

For data processing of the derived experimental responses which is essential for the development of the EVD model in the 4-components mixture optimization, quadratic (square-root, λ = 0.5) power transformation was adopted. The experimental responses obtained for CBR, UCS and RLS indicated 11–42%, 410–755 kN/m^2^ and 33–59% with max-to-min ratios of 3.81818, 1.84146 and 1.78788 respectively. Statistical analysis was carried out through fits summary, diagnostic computation and influences, numerical and graphical optimization to ascertain the optimal 4-components mixture ratio to improve the engineering behaviour of problematic soil. After the development of the model coefficients, Post analysis, point prediction, confirmation and model simulation were further achieved to validate the prediction performance of the EVD model with the aid of a design expert and Minitab 18 software.

### Fit-summary statistics

This statistical assessment is first executed on the system datasets to derive their viability and adequacy which would enable the actualization of the requirements for the EVD modelling measures using a mixture coding of L_Pseudo. The fit-summary computation provides important information which would aid the choice of starting point suitable for the development of the EVD model. The essential collection of statistical indicators such as lack-of-fit, coefficient-of-determination (R-sqd), sum-of-squares (SS) and predicted-sum-of-squares (PRESS) is calculated as presented in Tables 9, 10, 11, 12, 13, 14, 15, 16, 17. The computed statistical fit results indicated a preference for the quadratic model for CBR response with R-sqd. of 0.9929 while linear models were suggested for both UCS and RLS responses with R-sqd. of 0.8844 and 0.9463 respectively. From the sequential sum of squares, p-value of 0.0040, 0.0001 and 0.0001 were calculated for CBR, UCS and RLS responses respectively.

**Table 9:** Model summary statistics for CBR response.

Source	Std. Dev	R-Sqd	R-Sqd. Adjtd	R-Sqd. Pred	PRESS	
Linear	0.42	0.8569	0.8179	0.7154	3.94	
Quadratic	0.14	0.9929	0.9801	0.9110	1.23	Suggested
Special cubic	0.039	0.9999	0.9985	− 0.8270	25.26	
Cubic					+	Aliased

+ Case(s) with leverage of 1.0000: PRESS statistic not defined.

**Table 10:** Lack of fit for CBR response.

Source	Sequential *p*-value	Adjtd. R-Sqd	Pred. R-Sqd	
Linear	< 0.0001	0.8179	0.7154	
Quadratic	0.0040	0.9801	0.9110	Suggested
Special cubic	0.1850	0.9985	-0.8270	
Cubic				Aliased

**Table 11:** sequential Model sum of squares [Type I] for CBR response.

Source	SS	df	MS	F-Value	*p*-value Prob > F	
Mean versus total	386.17	1	386.17			
Linear versus mean	11.85	3	3.95	21.96	< 0.0001	
Quadratic versus linear	1.88	6	0.31	15.96	0.0040	Suggested
Sp cubic versus quadratic	0.097	4	0.024	16.01	0.1850	
Cubic vs Sp cubic	1.509E-003	1	1.509E-003			Aliased
Residual	0.000	0				
Total	400.00	15	26.67			

*SS* Sum-of-Squares; *MS* Mean-Square; *df* degree of freedom.

**Table 12:** Model Summary Statistics for UCS Response.

Source	Std. Dev	R-Sqd	R-Sqd. Adjtd	R-Sqd. Pred	PRESS	
Linear	0.91	0.8844	0.8528	0.7928	16.38	Suggested
Quadratic	0.53	0.9820	0.9496	0.7563	19.26	
Special Cubic	0.16	0.9997	0.9956	–4.2859	417.81	
Cubic					+	Aliased

**Table 13:** Lack of Fit for UCS Response.

Source	Sequential *p*-value	R-Sqd. Adjtd	R-Sqd. Pred	
Linear	< 0.0001	0.8528	0.7928	Suggested
Quadratic	0.0596	0.9496	0.7563	
Special Cubic	0.1975	0.9956	–4.2859	
Cubic				Aliased

**Table 14:** Sequential model sum of squares [Type I] for UCS response.

Source	SS	df	MS	F-Value	*p*-value Prob > F	
Mean versus total	8860.96	1	8860.96			
Linear versus mean	69.90	3	23.30	28.04	< 0.0001	Suggested
Quadratic versus linear	7.72	6	1.29	4.52	0.0596	
Sp Cubic versus quadratic	1.40	4	0.35	14.00	0.1975	
Cubic versus Sp cubic	0.025	1	0.025			Aliased
Residual	0.000	0				
Total	8940.00	15	596.00

**Table 15:** Model summary statistics for RLS Response.

Source	Std. Dev	R-Sqd	R-Sqd. Adjtd	R-Sqd. Pred	PRESS	
Linear	0.19	0.9463	0.9317	0.9019	0.72	Suggested
Quadratic	0.23	0.9628	0.8957	0.5329	3.41	
Special cubic	0.057	0.9995	0.9937	–6.5653	55.29	
Cubic					+	Aliased

**Table 16:** Lack of Fit for RLS response.

Source	Sequential *p*-value	R-Sqd. Adjtd	R-Sqd. Pred	
Linear	< 0.0001	0.9317	0.9019	Suggested
Quadratic	0.8718	0.8957	0.5329	
Special Cubic	0.1646	0.9937	− 6.5653	
Cubic				Aliased

**Table 17:** Sequential Model Sum of Squares [Type I] for RLS Response.

Source	SS	df	MS	F-Value	*p*-value Prob > F	
Mean versus total	651.69	1	651.69			
Linear versus mean	6.92	3	2.31	64.61	< 0.0001	Suggested
Quadratic versus linear	0.12	6	0.020	0.37	0.8718	
Sp Cubic versus quadratic	0.27	4	0.067	20.34	0.1646	
Cubic versus Sp cubic	3.304E − 003	1	3.304E − 003			Aliased
Residual	0.000	0				
Total	659.00	15	43.93			

### Analysis of variance (ANOVA) statistical results

Analysis of variance statistical computation was carried out for the preferred model to assess the mixture ingredients fractions’ significance levels on the CBR, UCS and RLS response variables using L_Pseudo coded units. The ANOVA computation of partial sum-of-squares and R-sqd. Statistics calculation results are presented in Tables 18, 19, 20, 21, 22, 23. From the statistical analysis results, the model p-value of 0.0001 was derived for the three response variables while F-value of 77.67, 28.04, and 64.61 were obtained for CBR, UCS and RLS responses respectively. The indication of this statistical result showed that the derived model terms are statistically significant.

**Table 18:** ANOVA for quadratic mixture model for CBR response.

*** Mixture component coding is L_Pseudo. ***						
Analysis of variance table [Partial sum of squares—Type III]						
Source	SS	df	MS	F-Value	p-value Prob > F	
Model	13.73	9	1.53	77.67	< 0.0001	significant
^1^Linear Mixture	11.85	3	3.95	201.09	< 0.0001	
AB	0.21	1	0.21	10.51	0.0229	
AC	0.17	1	0.17	8.66	0.0321	
AD	0.11	1	0.11	5.51	0.0658	
BC	0.44	1	0.44	22.21	0.0053	
BD	0.073	1	0.073	3.74	0.1109	
CD	4.222E − 004	1	4.222E-004	0.021	0.8892	
Residual	0.098	5	0.020			
Cor Total	13.83	14				

^1^linear mixtures inferences uses SS Type I.

**Table 19:** R-sqd. calculations for CBR response.

Std. Dev	0.14		R-Sqd	0.9929
Mean	5.07		R-Sqd. Adjtd	0.9801
C.V. %	2.76		R-Sqd. Pred	0.9110
PRESS	1.23		Adeq.-Prec	27.804
− 2 Log Likelihood	− 32.86		BIC	− 8.49
			AICc	21.14

**Table 20:** ANOVA for linear mixture model for UCS response.

*** Mixture component coding is L_Pseudo. ***						
Analysis of variance table [Partial sum of squares—Type III]						
Source	SS	df	MS	F-Value	p-value Prob > F	
Model	69.90	3	23.30	28.04	< 0.0001	significant
^1^*Linear mixture*	*69.90*	*3*	*23.30*	*28.04*	< *0.0001*	
Residual	9.14	11	0.83			
Cor Total	79.04	14				

^1^linear mixtures inferences uses SS Type I.

**Table 21:** R-sqd. calculations for UCS response.

Std. Dev	0.91		R-Squared	0.8844
Mean	24.30		R-Sqd. Adjtd	0.8528
C.V. %	3.75		R-Sqd. Pred	0.7928
PRESS	16.38		Adeq.-Prec	14.730
-2 Log Likelihood	35.14		BIC	43.26
			AICc	43.32

**Table 22:** ANOVA for Linear Mixture model for RLS Response.

*** Mixture component coding is L_Pseudo. ***						
Analysis of variance table [Partial sum of squares—Type III]						
Source	**SS**	df	MS	F-Value	*p*-value Prob > F	
Model	6.92	3	2.31	64.61	< 0.0001	significant
^1^*Linear Mixture*	*6.92*	*3*	*2.31*	*64.61*	< *0.0001*	
Residual	0.39	11	0.036			
Cor Total	7.31	14				

^1^linear mixtures inferences uses SS Type I.

**Table 23:** R-sqd. calculations for RLS response.

Std. Dev	0.19		R-Squared	0.9463
Mean	6.59		R-Sqd. Adjtd	0.9317
C.V. %	2.87		R-Sqd. Pred	0.9019
PRESS	0.72		Adeq.-Prec	22.772
− 2 Log Likelihood	–12.08		BIC	–3.96
			AICc	–3.90

The calculated 0.9110 result for R-Sqd. Pred. showed satisfactory agreement with the R-Sqd. Adjtd. of 0.9801 which indicates a difference of less than 0.2 when both indices were compared. Adeq.-Prec. evaluates the signal-to-noise ratio and a ratio value of 27.804 was obtained which signified an adequate signal with a computed ratio outcome > 4.

The computed 0.7928 result for R-Sqd. Pred. implied suitable agreement with the R-Sqd. Adjtd. of 0.8528 which indicates a difference of < 0.2 when both statistical indices were compared. Adeq.-Prec. measures the signal-to-noise ratio and a ratio value of 14.730 was obtained which denoted an adequate signal with a computed ratio outcome > 4.

The calculated 0.9019 results for R-Sqd. Pred. implied suitable agreement with the R-Sqd. Adjtd. of 0.9317 which indicates a difference of < 0.2 when both statistical measures were compared. Adeq.-Prec. assesses the signal-to-noise ratio and a ratio value of 22.772 was derived which denoted an adequate signal with a computed ratio outcome greater than 4.

### Coefficient estimates and model equations

After ANOVA computation which involved thorough statistical assessments were executed to achieve suitable estimates of the model coefficients for the three response parameters carried out with the aid of Design Expert software. The calculations show low and high confidence intervals for the model estimates, variance inflation factor (VIF), standard error and derived model numerical coefficients as presented in Tables 24, 25, 26, 27, 28, 29, 30, 31, 32, 33, 34. The lack of orthogonality impact on the variances of the EVD model coefficients developed is assessed through VIF which is proportional to the square of the model’s standard error. The model coefficients for the CBR responses which were derived using quadratic polynomials and UCS and RLS derived using linear polynomials are presented in Tables 25, 30 and 34 respectively. The coefficients for the UCS and RLS responses were linear polynomial, hence, base point terms and constraint region bounded factor level effects for Piepel and Cox direction were analyzed as presented in Tables 27, 28, 29, 32, 33. The derived results for UCS responses in Piepel direction showed Prob >|t| of 0.2004, 0.0005, < 0.0001 and 0.0268, for components A, B, C and D respectively and 0.4912, 0.2406, < 0.0001 and 0.0554 for components A, B, C and D respectively in Cox direction. Therefore, results for RLS responses in Piepel direction indicated Prob >|t| of 0.0026, < 0.0001, < 0.0001 and 0.0069 for components A, B, C and D respectively while 0.0148, 0.2503, < 0.0001, 0.0368 for components A, B, C and D respectively in Cox direction.

**Table 24:** Quadratic Model Coefficients Calculation Results for CBR Response.

Component	Coefficient estimate	df	Standard error	95% CI low	95% CI high	VIF
A-Water	21.36	1	5.96	6.03	36.69	574.62
B-TWD	4.58	1	0.20	4.06	5.11	6.56
C-CK	6.65	1	0.21	6.11	7.19	7.29
D-Soil	5.12	1	2.22	− 0.58	10.81	177.51
AB	− 24.90	1	7.68	− 44.64	− 5.15	160.86
AC	− 22.78	1	7.74	− 42.68	− 2.88	170.15
AD	− 18.25	1	7.78	− 38.24	1.73	45.52
BC	2.77	1	0.59	1.26	4.28	2.48
BD	− 6.47	1	3.35	− 15.07	2.13	53.73
CD	− 0.50	1	3.42	− 9.29	8.29	64.91

**Table 25:** Derived final equation for CBR response.

L_Pseudo Coding	* A	* B	* C	* D	* AB	* AC	* AD	* BC	* BD	* CD
Sqrt. (CBR-soaked)	21.36	4.58	6.65	5.12	− 24.90	− 22.78	− 18.25	2.77	− 6.47	− 0.50

**Table 26:** Linear model coefficients calculation results for UCS response.

Component	Coefficient estimate	df	Standard error	95% CI low	95% CI high	VIF
A-Water	21.32	1	2.27	16.31	26.32	1.97
B-TWD	21.81	1	0.63	20.42	23.20	1.51
C-CK	29.30	1	0.65	27.86	30.73	1.64
D-Soil	20.67	1	1.46	17.46	23.89	1.82

**Table 27:** UCS and RLS responses base points in terms of pseudo and real components.

Coding	A	B	C	D
Pseudo	+ 0.095238	+ 0.380952	+ 0.380952	+ 0.142857
Real	+ 0.142857	+ 0.221429	+ 0.221429	+ 0.414286

**Table 28:** Constraint region bounded component effects for piepel direction for UCS response.

Component	Gradient in reals	Component effect	Gradient Std. error	Approx. t for H_0_ gradient = 0	Prob >|t|	Gradient in pseudo
A-Water	− 7.70	− 0.77	5.65	− 1.36	0.2004	− 3.46
B-TWD	− 9.49	− 3.79	1.94	− 4.88	0.0005	− 4.27
C-CK	17.39	6.96	2.04	8.52	< 0.0001	7.82
D-Soil	− 9.80	− 1.47	3.84	− 2.55	0.0268	− 4.41

**Table 29:** Constraint region bounded component effects for cox direction for UCS response.

Component	Gradient in reals	Component effect	Gradient Std. error	Approx. t for H_0_ gradient = 0	Prob >|t|
A-Water	− 4.17	− 0.42	5.85	− 0.71	0.4912
B-TWD	− 3.19	− 0.90	2.57	− 1.24	0.2406
C-CK	18.18	5.13	2.69	6.76	< 0.0001
D-Soil	− 8.55	− 1.28	3.99	− 2.14	0.0554

**Table 30:** derived Final equation for UCS response.

Coding	*A	*B	*C	*D
L_Pseudo	+ 21.32	+ 21.81	+ 29.30	+ 20.67

**Table 31:** Linear Model Coefficients Calculation Results for RLS Response.

Component	Coefficient estimate	df	Standard error	95% CI low	95% CI high	VIF
A-Water	4.80	1	0.47	3.77	5.84	1.97
B-TWD	5.93	1	0.13	5.64	6.21	1.51
C-CK	8.22	1	0.14	7.92	8.52	1.64
D-Soil	5.64	1	0.30	4.97	6.30	1.82

**Table 32:** Constraint egion bounded component effects for Piepel direction for RLS response.

Component	Gradient in reals	Component effect	Gradient Std. error	Approx. t for H_0_ gradient = 0	Prob >|t|	Gradient in pseudo
A-Water	− 4.54	− 0.45	1.17	− 3.88	0.0026	− 2.04
B-TWD	− 2.60	− 1.04	0.40	− 6.46	< 0.0001	− 1.17
C-CK	5.63	2.25	0.42	13.31	< 0.0001	2.53
D-Soil	− 2.63	− 0.40	0.80	− 3.31	0.0069	− 1.19

**Table 33:** Constraint region bounded component effects for cox direction for RLS response.

Component	Gradient in reals	Component effect	Gradient Std. error	Approx. t for H_0_ gradient = 0	Prob >|t|
A-Water	− 3.50	− 0.35	1.21	− 2.89	0.0148
B-TWD	− 0.65	− 0.18	0.53	− 1.21	0.2503
C-CK	5.90	1.66	0.56	10.58	< 0.0001
D-Soil	− 1.96	− 0.29	0.83	− 2.38	0.0368

**Table 34:** Derived final equation for UCS response.

Coding	*A	*B	*C	*D
L_Pseudo	+ 4.80	+ 5.93	+ 8.22	+ 5.64

### Diagnostics plots

To achieve the corroboration of the regression model assumptions and to elucidate if the experimental observations utilized for the EVD-model development were significantly unjustifiable and at the same time pose a noticeable influence on the analysis results. Studentized residuals were adopted to accomplish this statistical analysis which is used to detect outliers which significantly differ from regular observations as a result of a verifiable error in the mixture design. Moreover, varying normal distributions observed are mapped to a unit standard normal-distribution using the studentized residuals for the CBR, UCS and RLS responses respectively. Statistical-diagnostic tests carried out include predicted vs. residual, normal probability test, predicted vs. actual, run plot vs. residuals, and box-cox power transformation.

#### Graph of normal-probability

**Figure 10:**
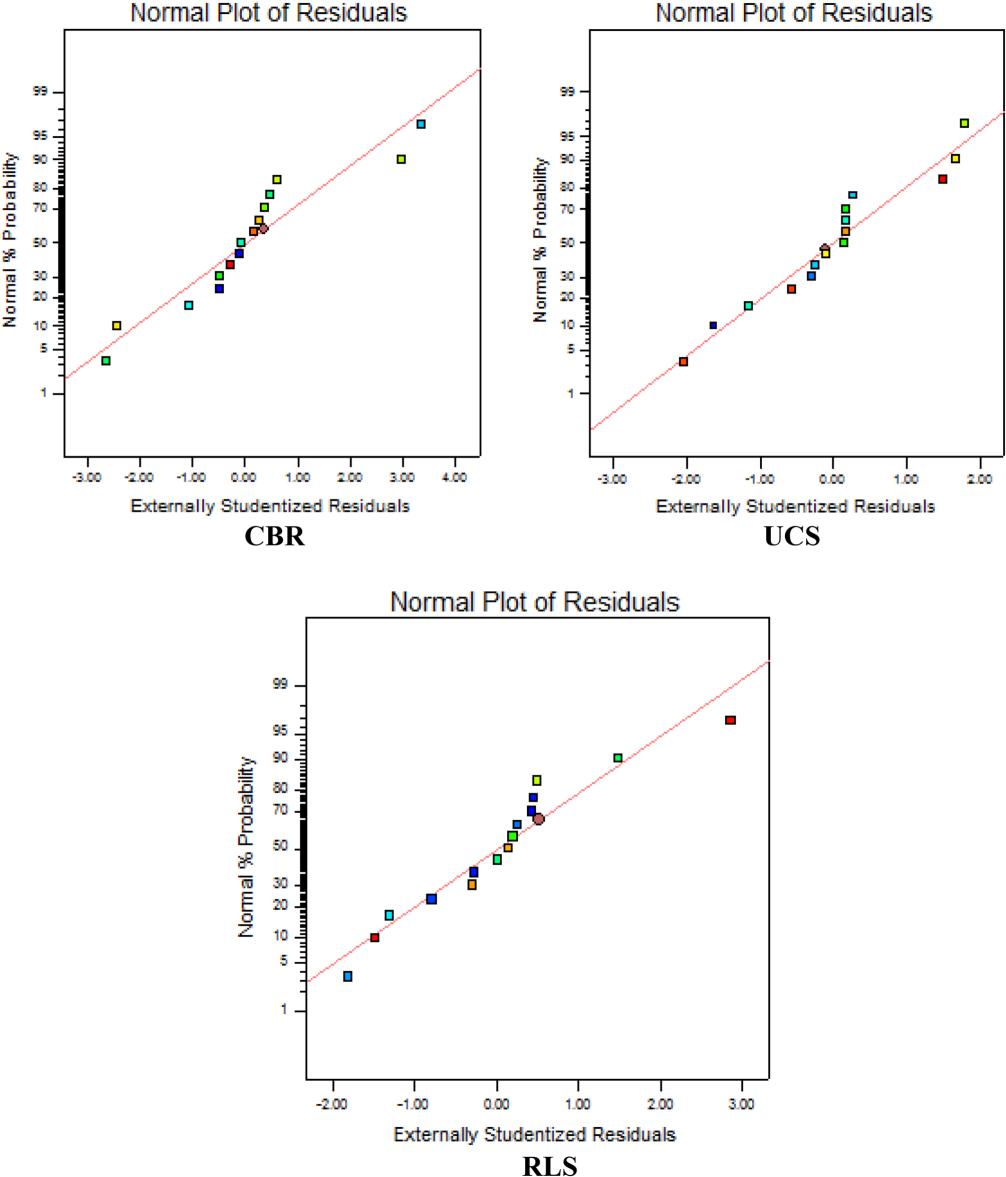
Graphical presentation of residuals normal Probability.

The graphical results evaluating the normal probability help to confirm that the derived errors also known as residuals follow or assume normal distribution positioned nearest to the regression line. The plot illustrates the relationship between the externally studentized residuals on the x-axis against the normal probability (%) on the y-axis for the CBR, UCS and RLS response variables as shown in Fig. 10. Details such as definite configurations like curvilinear shapes are examined carefully to ascertain the possibility of deploying suitable model transformation of the response parameters. The results derived from the plots indicate the majority of the points from the horizontal axis are within the range of -1 to + 1 studentized residuals and also within 10–90% normal probability for the three response cases under study.

#### Graph of predicted versus studentized residuals

**Figure 11:**
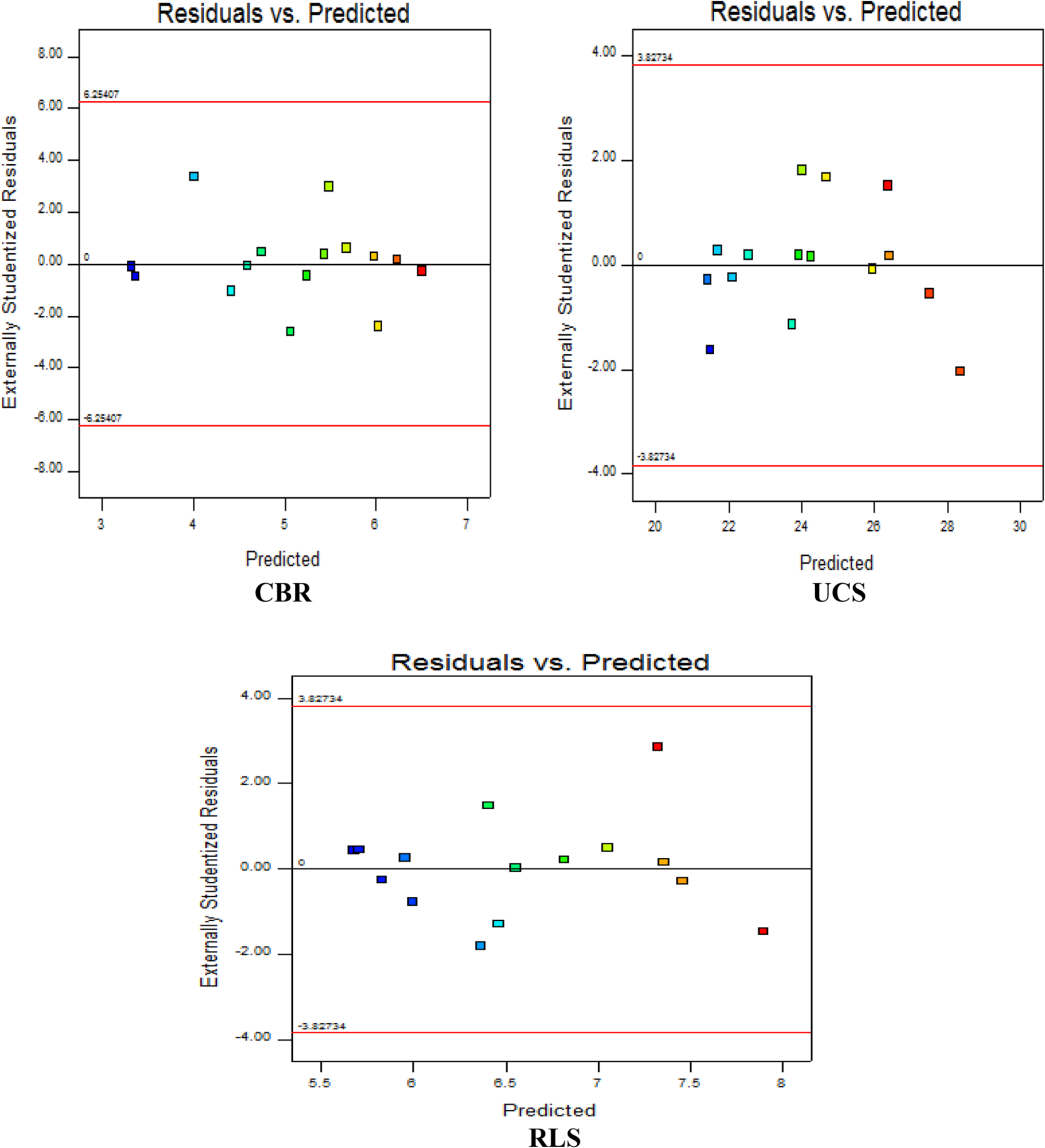
Diagnostic statistical graphs of residual versus predicted results.

The constant variance assumption of the residuals is validated for the three response parameters through the residuals vs. predicted statistical diagnostic test. The plot presents the model-predicted values on the x-axis against the externally studentized residuals on the y-axis of the graph as shown in Fig. 11. The presented graphical results indicated clustering of the derived model-predicted values at the zero studentized point for the majority of the observations. The plot for CBR response was observed to be positioned at the limits of ± 4 within the boundary points of ± 6.254, the responses for UCS and RLS were thus observed to show a similar trend as they are situated at ± 2 limits within the boundary points of ± 3.8273.

#### Experimental run versus studentized residuals Plot

**Figure 12:**
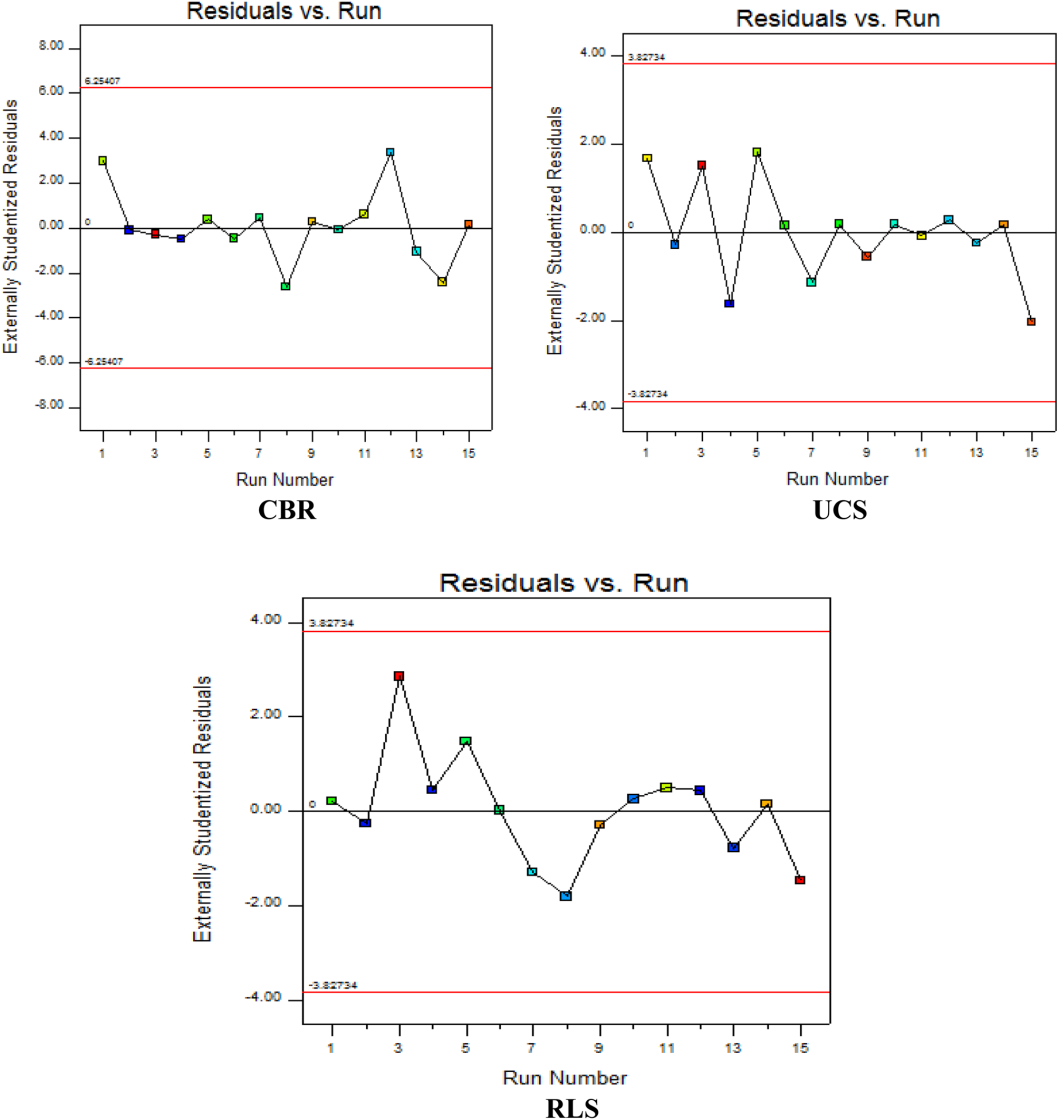
Residual versus experimental run diagnostic graphs.

The examination for plundering factors which pose a significant impact on the response variables during the statistical calculations is achieved using this diagnostic computation graph. The plot presents the experimental run’s effects on the target responses on the x-axis against the externally studentized residuals on the y-axis as shown in Fig. 12. From the graphical results similar to the residual vs. predicted diagnostic plots, the observed design points were situated at the boundary points of ± 4 for the CBR response and ± 2 for both the UCS and RLS response which signifies a time-dependent lurking variable in the background of the presented scattered plot.

#### Predicted versus experimental result graphical plots

**Figure 13:**
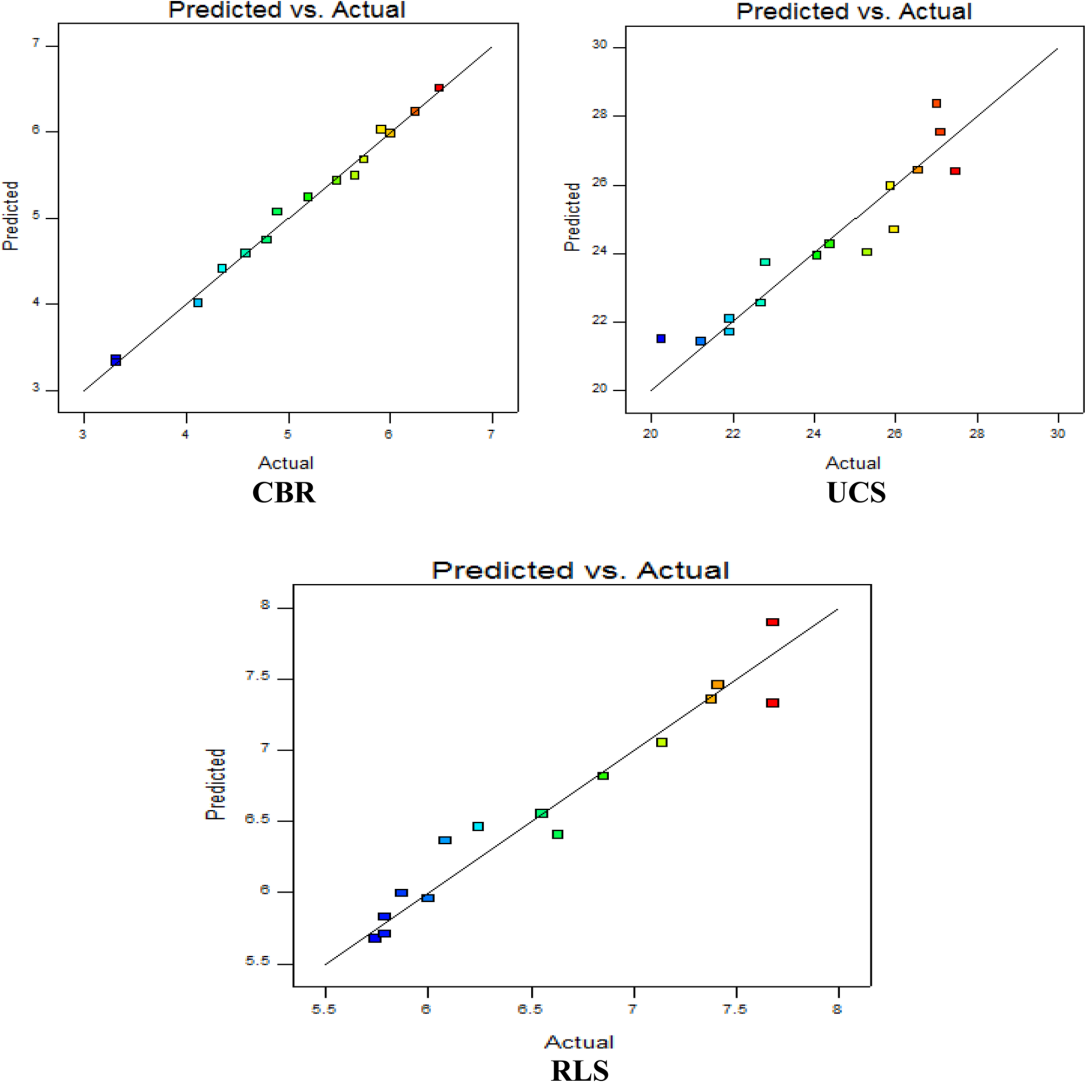
Diagnostic graphs of actual versus model predicted.

This diagnostic statistical analysis plot essentially helps to obtain a unitary or group of values which are difficult to accurately estimate by the generated EVD-model in a straight line graph of the laboratory outcomes on the x-axis and the predicted values on the y-axis as shown in Fig. 13. The essence of this diagnostic computation is to assess the relationships between the square root transformed model predicted and actual values by how well the designed experimental data fit the regression line. The details derived from the graphical plots for the three responses indicated a good and acceptable correlation between the model estimated and laboratory values.

#### Power transformation using box-cox plot

**Figure 14:**
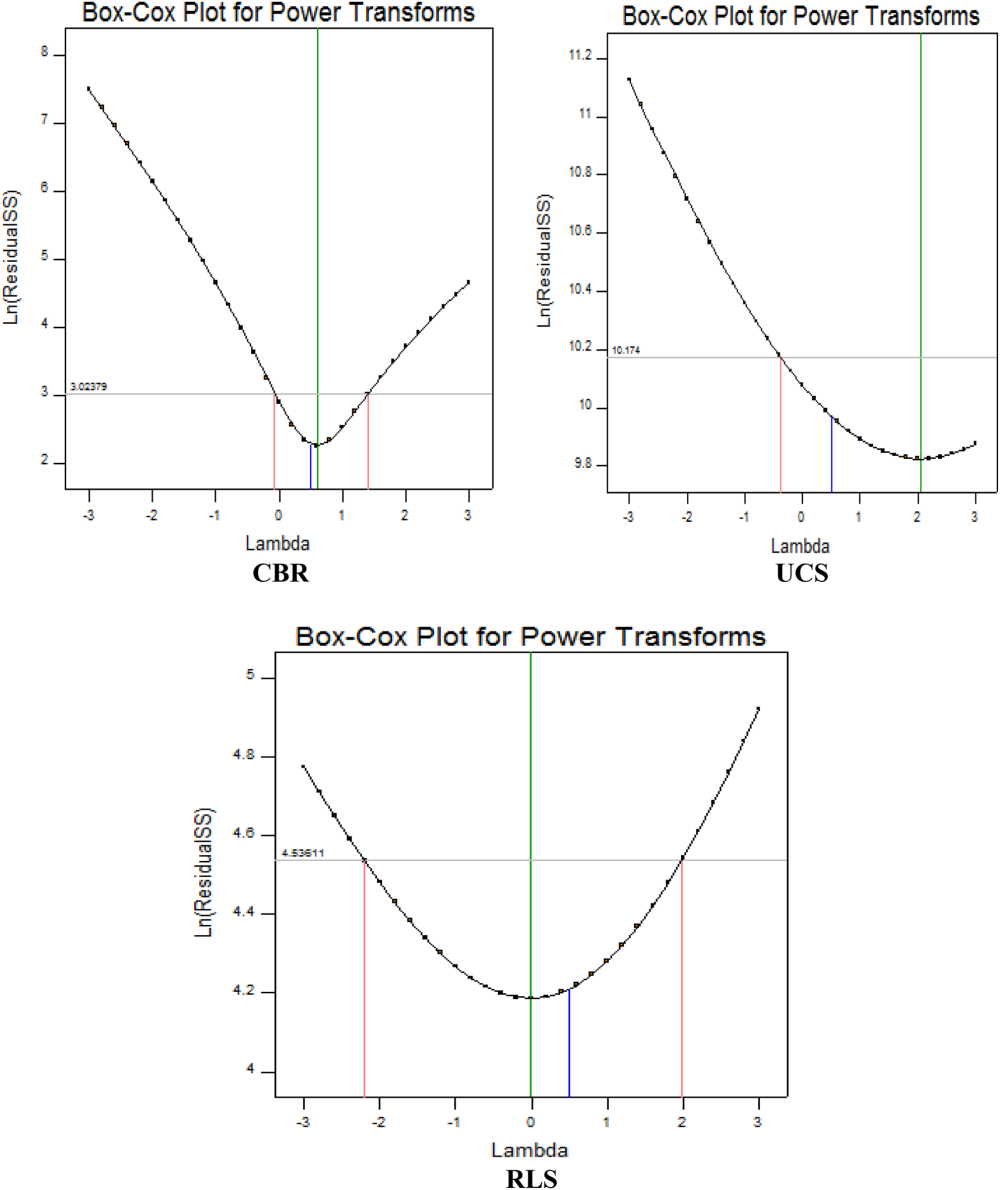
Box-Cox plots for power transformation.

This analytical computation plot offers suitable guidelines for the selection of an adequate power transformation model to examine the significant impacts on the response parameters by the factor levels. The results derived from this diagnostic assessment help to attain the best lambda outcomes on the horizontal axis of the plot which are derived at the minimum point of the parabolic curve corresponding to the sum of squares residuals’ natural logarithm on the vertical axis as shown in Fig. 14. From the presented graphs for CBR response showed current lambda of 0.5, confidence intervals (C.I.) of -0.07 and 1.41 lambda for low and high limits respectively, and best lambda of 0.6. Also, the UCS response showed current and best lambda of 0.5 and 2.06 respectively, low and high C.I. of -0.38 and 4.55 respectively while the RLS response showed current and best lambda of 0.5 and -0.02 respectively, low and high C.I. of -2.2 and 1.98 respectively.

### Diagnostic-influence plots

The statistical diagnostic influence evaluates the significant impact of runs of the experiment on the outcome of the analysis. The influence plots present a suitable statistical perspective in the experimental design when multiple or unitary cases stands out from other groups and the statistical influence computation is achieved using the following analytical tools; leverage vs. the experimental run, cook’s distance and statistical difference in fits (DFFITS) vs. run of experiments.

#### The cook’s distance

**Figure 15:**
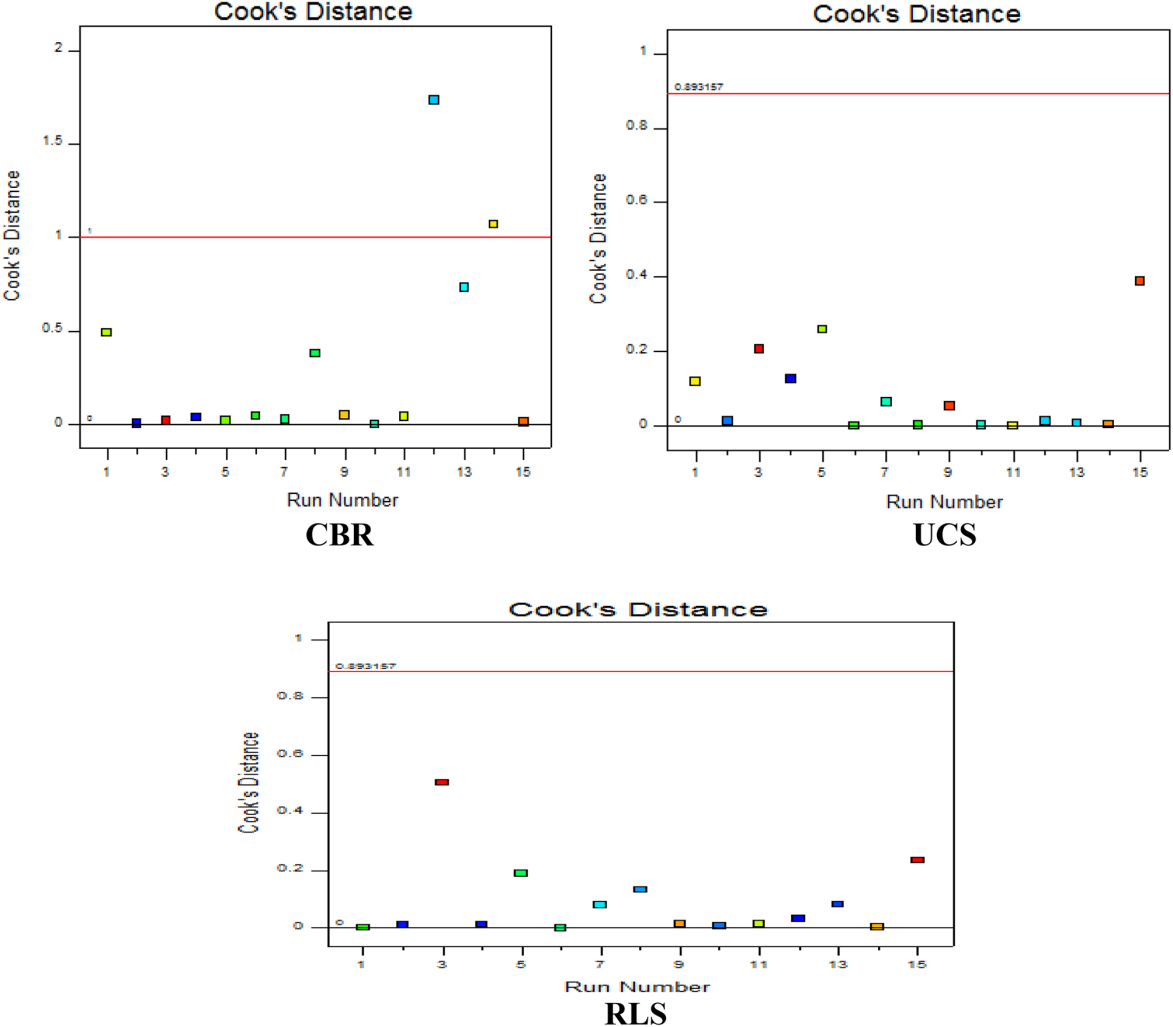
Cook’s distance.

The Cook’s distance is adopted in regression analysis to ascertain the influence of data points or outliers in explanatory variables concerning the target response variables. This computation also helps to determine the feasible experimental planes where negative effects are achieved and also where acceptable correlation outcomes are derived. The plot presents runs of experiments on the x-axis against the calculated cook’s distance result as shown in Fig. 15. From the derived results, the experimental runs were mostly positioned at the cook distance zero point, however, for the CBR response, the experimental points lie within the cook’s distance limits of 0–2 and 0–1 for both the UCS and RLS responses.

#### Leverage versus run

**Figure 16:**
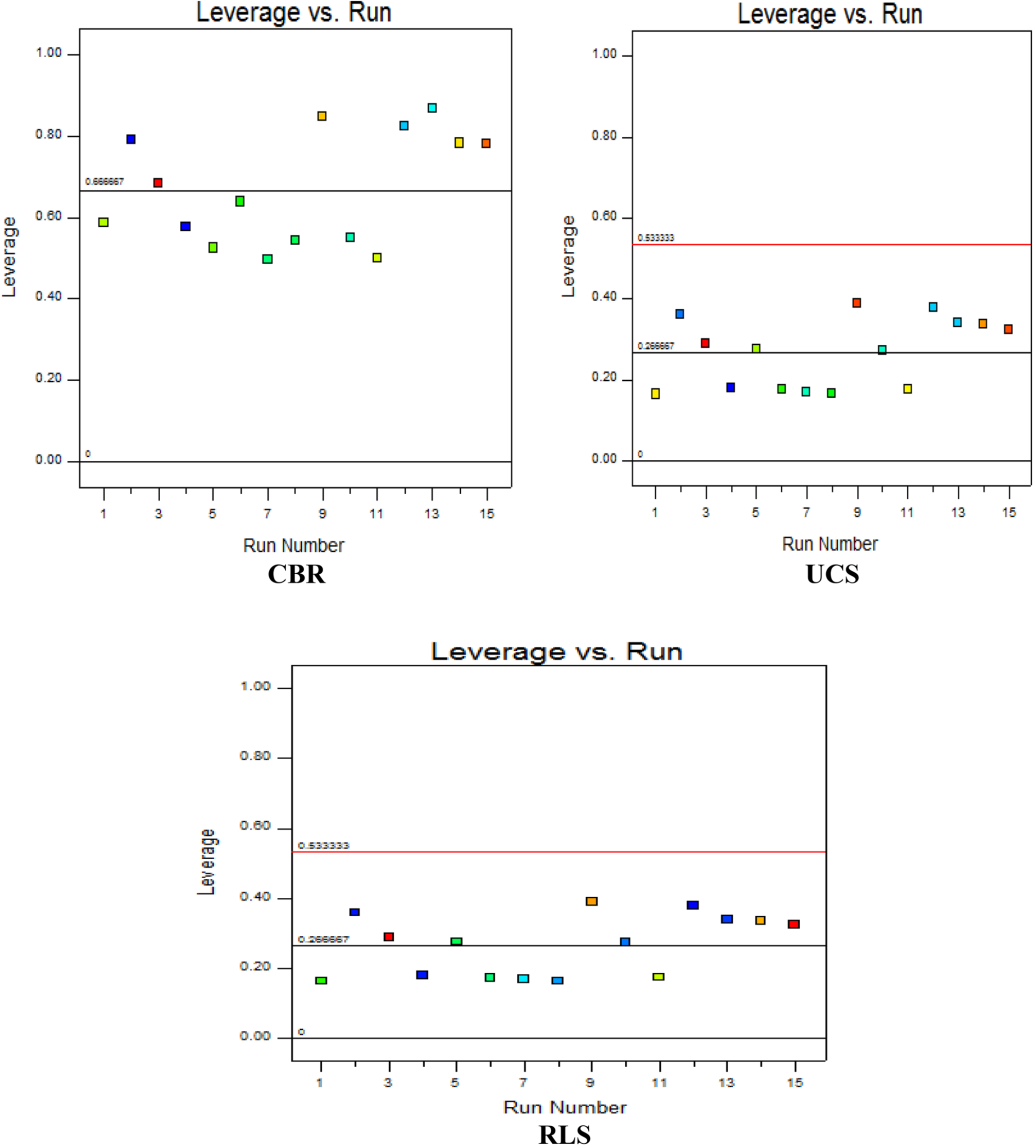
Diagnostic influence plot of leverage versus run.

Leverage computation is a statistical diagnostic influence tool which is utilized to examine the extents to which each experimental point affects the developed model’s goodness-of-fit. It is also deployed to evaluate the distance with which the explanatory variables from a particular observation are far apart from the set of other observations in the mixture design experiments. This diagnostic influence plot presents the derived leverage values from 0 to 1 on the vertical axis against the run of experiments on the horizontal axis as shown in Fig. 16. The presented plots for the CBR response lie within leverage values of 0.5–0.9 with the boundary line drawn at 0.6667. However, the UCS and RLS responses exhibited similar trends and lie within the leverage points of 0.2–0.4 with the boundary lines observed at 0.53333–0.26667.

#### Statistical difference in fits (DFFITS) versus runs

**Figure 17:**
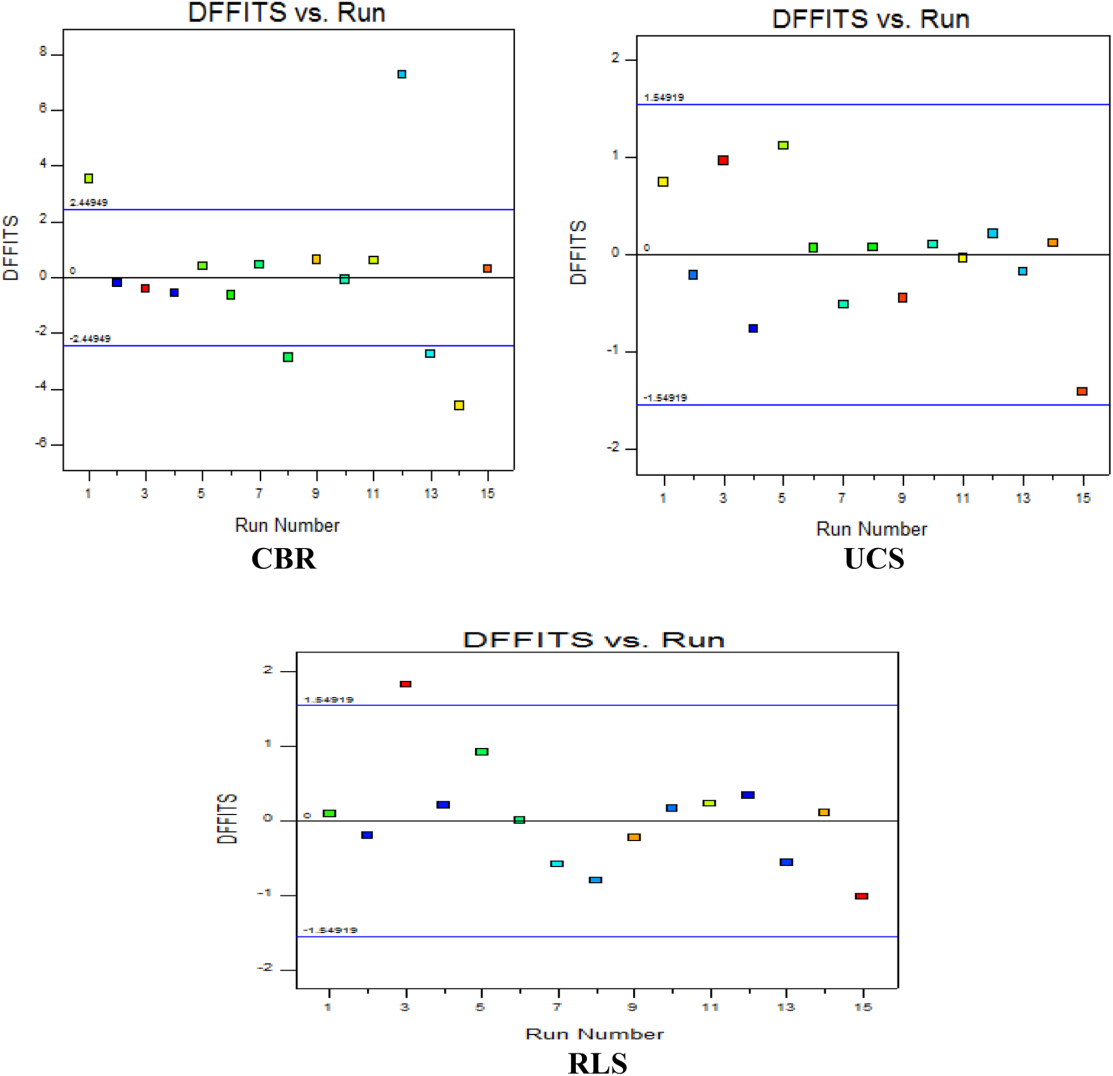
Diagnostic influence plot of dffits versus experimental run.

Statistical Difference in Fits (DFFITS) is a statistical influence technique which evaluates the variation of the EVD-model predicted response for the ith observation in the experimental mixture design. This diagnostic test helps to clarify for a particular design point the estimated model variability when it is barred for the model fitting processes as shown in Fig. 17. The presented analytical plots which indicate the fifteen design experimental runs on the horizontal axis against the computed DFFITS outcomes on the y-axis. From the results, the majority of the data points were observed to be positioned within the boundary points of ± 2.4449 except runs number 1, 8, 12, 13 and 14 for the CBR response. However, the data points for both CBR and RLS were observed to be situated within similar boundary points of ± 1.54919 except experimental run number 1 for RLS response. The diagnostic statistical summary and influences for the analytical computations carried out in this experimental investigation and EVD model development for the CBR and UCS responses are shown in Tables 35, 36, 37. The results present the predicted vs. actual values, the computed residuals, leverages, internally and externally studentized residuals, cook’s distance and influence on fitted value.

**Table 35:** Diagnostics case statistics for CBR response.

Run order	Actual value	Predicted value	Residual	Leverage	Internally studentized residual	Externally studentized residual	Cook's distance	Influence on fitted value DFFITS	Standard order
7	4.80	4.74	0.051	0.496	0.515	0.473	0.026	0.469	15
14	5.92	6.03	− 0.11	0.783	− 1.723	− 2.419	1.070^1^	− 4.592^1^	14
2	3.32	3.32	− 7.244E− 003	0.792	− 0.113	− 0.101	0.005	− 0.198	13
10	4.58	4.59	− 8.325E− 003	0.551	− 0.089	− 0.079	0.001	− 0.088	12
5	5.48	5.44	0.040	0.525	0.417	0.380	0.019	0.400	11
11	5.74	5.68	0.065	0.501	0.656	0.614	0.043	0.614	10
4	3.32	3.36	− 0.048	0.576	− 0.522	− 0.480	0.037	− 0.559	9
1	5.66	5.49	0.17	0.587	1.856	2.978	0.490	3.551^1^	8
8	4.90	5.07	− 0.17	0.545	− 1.779	− 2.628	0.379	− 2.875^1^	7
15	6.24	6.23	0.012	0.781	0.180	0.161	0.012	0.305	6
13	4.36	4.41	− 0.053	0.869	− 1.048	− 1.061	0.730	− 2.736^1^	5
6	5.20	5.24	− 0.043	0.639	− 0.513	− 0.471	0.047	− 0.627	4
9	6.00	5.98	0.017	0.847	0.302	0.273	0.051	0.642	3
3	6.48	6.51	− 0.025	0.683	− 0.311	− 0.281	0.021	− 0.412	2
12	4.12	4.01	0.11	0.824	1.921	3.359	1.732^1^	7.276^1^	1

^1 Exceeds limits.

**Table 36:** Diagnostics case statistics for UCS response.

Run order	Actual value	Predicted value	Residual	Leverage	Internally studentized residual	Externally studentized residual	Cook's distance	Influence on fitted value dffits	Standard order
7	22.80	23.74	− 0.93	0.169	− 1.125	− 1.140	0.064	− 0.515	15
14	26.55	26.42	0.13	0.337	0.175	0.167	0.004	0.119	14
2	21.21	21.43	− 0.22	0.360	− 0.298	− 0.286	0.013	− 0.214	13
10	22.69	22.55	0.14	0.274	0.184	0.176	0.003	0.108	12
5	25.30	24.02	1.28	0.277	1.647	1.809	0.260	1.120	11
11	25.88	25.96	− 0.072	0.175	− 0.087	− 0.083	0.000	− 0.038	10
4	20.25	21.50	− 1.26	0.180	− 1.520	− 1.631	0.127	− 0.764	9
1	25.98	24.69	1.29	0.164	1.552	1.674	0.118	0.742	8
8	24.08	23.93	0.15	0.165	0.186	0.177	0.002	0.079	7
15	27.02	28.36	− 1.35	0.324	− 1.797	− 2.038	0.388	− 1.412	6
13	21.91	22.10	− 0.19	0.340	− 0.254	− 0.243	0.008	− 0.174	5
6	24.39	24.27	0.13	0.175	0.152	0.145	0.001	0.067	4
9	27.11	27.52	− 0.41	0.390	− 0.580	− 0.561	0.054	− 0.449	3
3	27.48	26.38	1.09	0.290	1.426	1.505	0.208	0.962	2
12	21.91	21.70	0.21	0.380	0.290	0.277	0.013	0.217	1

**Table 37:** Diagnostics case statistics for RLS response.

Run order	Actual value	predicted value	Residual	Leverage	Internally Studentized residual	Externally Studentized residual	Cook's Distance	Influence on fitted value DFFITS	Standard order
7	6.24	6.46	− 0.22	0.169	− 1.256	− 1.294	0.080	− 0.584	15
14	7.38	7.36	0.024	0.337	0.157	0.150	0.003	0.107	14
2	5.79	5.83	− 0.042	0.360	− 0.277	− 0.265	0.011	− 0.199	13
10	6.00	5.96	0.044	0.274	0.273	0.261	0.007	0.160	12
5	6.63	6.41	0.23	0.277	1.410	1.485	0.190	0.920	11
11	7.14	7.05	0.088	0.175	0.515	0.498	0.014	0.229	10
4	5.79	5.71	0.081	0.180	0.471	0.454	0.012	0.213	9
1	6.86	6.82	0.037	0.164	0.217	0.207	0.002	0.092	8
8	6.08	6.37	− 0.28	0.165	− 1.644	− 1.804	0.133	− 0.801	7
15	7.68	7.90	− 0.22	0.324	− 1.399	− 1.471	0.235	− 1.019	6
13	5.87	6.00	− 0.12	0.340	− 0.797	− 0.783	0.082	− 0.561	5
6	6.56	6.55	2.722E− 003	0.175	0.016	0.015	0.000	0.007	4
9	7.42	7.46	− 0.044	0.390	− 0.299	− 0.286	0.014	− 0.228	3
3	7.68	7.33	0.35	0.290	2.224	2.859	0.505	1.827^1^	2
12	5.74	5.68	0.068	0.380	0.454	0.437	0.032	0.342	1

^1^Exceeds limits.

### Optimization overview

The numerical and graphical optimization exercise is executed after the model fit computation, ANOVA, statistical diagnostics and influence calculations were carried out. The mixture experiment design optimization is achieved through the desirability function which examines the imposed components criteria or constraints upon the model variables to maximize the target responses. The criteria also known as the goal of the optimization can be minimum, maximum or in-range factors to constrain the variables based on the perceived objectives for the exercise. The objective functions are systematically adjusted using assigning adaptive weight functions in line with the preset criteria of the model variables. These multi-collinearity conditions would enable the attainment of favourable conditions to obtain a desirability score of 1.0 through a scale of $0 \le d\left( {y_{i} } \right) \le 1$ boundary conditions. The optimization measures for this 4-component mixture design explore the varying formulated components’ fractions combinations in the factor space to sort after response that the imposed criteria or components constraints for the factor levels and the corresponding optimized dependent variables simultaneously. The expected goal of this optimization exercise is set to maximize the target responses while the four mixture components are set at the in-range option to ascertain the optimal proportion of the factor levels to produce maximum responses bounded by the derived upper and lower limits from the experimental details as shown in Table 38.

**Table 38:** Variables constraints for optimization.

Name	Goal	Lower limit	Upper limit	Lower weight	Upper weight	Importance
A:Water	in-range	0.1	0.2	1	1	3
B:TWD	in-range	0.05	0.45	1	1	3
C:CK	in-range	0.05	0.45	1	1	3
D:Soil	in-range	0.35	0.5	1	1	3
CBR-soaked	max	11	42	1	1	3
UCS-7 days	max	410	755	1	1	3
RLS	max	33	59	1	1	3

The computed optimization solution derived from analytical proceedings of the 4-component mixture experiment design showing seventeen optimized solutions is presented in Table 39. From the derived results, solution number 13 was preferentially selected showing an optimal desirability score of 1.0 at a combination ratio of 0.100:0.097:0.450:0.0353 for water, TWD, CK and soil respectively to produce maximized CBR, UCS and RLS response of 44.611%, 810.687kN/m^2^ and 63.448% respectively.

**Table 39:** Computed mixture design optimization solutions.

Number	Water	TWD	CK	Soil	CBR-soaked	UCS-7 days	RLS	Desirability	
1	0.100	0.145	0.405	0.350	44.601	769.256	59.897	1.000	
2	0.110	0.087	0.450	0.353	42.135	809.986	63.031	1.000	
3	0.100	0.123	0.427	0.350	44.817	789.744	61.649	1.000	
4	0.100	0.132	0.418	0.350	44.751	781.442	60.939	1.000	
5	0.100	0.105	0.445	0.350	44.862	806.289	63.066	1.000	
6	0.100	0.071	0.450	0.379	42.601	806.961	63.181	1.000	
7	0.100	0.155	0.395	0.350	44.441	759.896	59.097	1.000	
8	0.110	0.133	0.407	0.350	42.305	770.221	59.652	1.000	
9	0.110	0.103	0.437	0.350	42.364	798.139	62.010	1.000	
10	0.107	0.114	0.429	0.350	43.201	791.279	61.555	1.000	
11	0.100	0.079	0.450	0.371	43.107	808.013	63.256	1.000	
12	0.109	0.122	0.419	0.350	42.504	781.409	60.617	1.000	
13	0.100	0.097	0.450	0.353	44.611	810.687	63.448	1.000	Selected
14	0.100	0.050	0.450	0.400	41.391	803.889	62.961	0.993	
15	0.190	0.050	0.410	0.350	35.411	768.172	56.830	0.897	
16	0.100	0.275	0.275	0.350	39.839	653.805	50.067	0.756	
17	0.150	0.250	0.250	0.350	30.932	629.978	46.561	0.598	

#### Graphical optimization plots of ramps and bar

**Figure 18:**
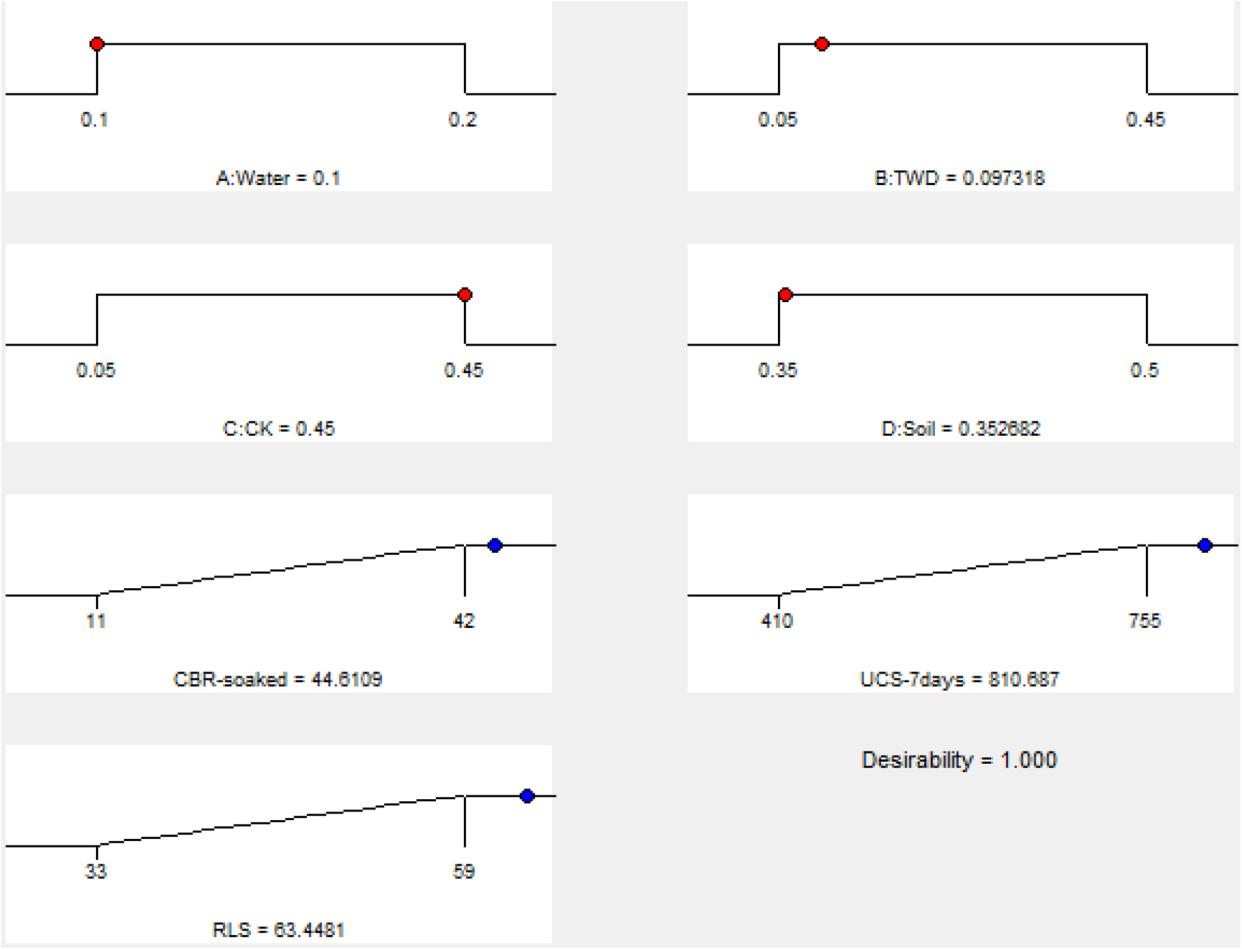
Ramps graph for the desirability function.

**Figure 19:**
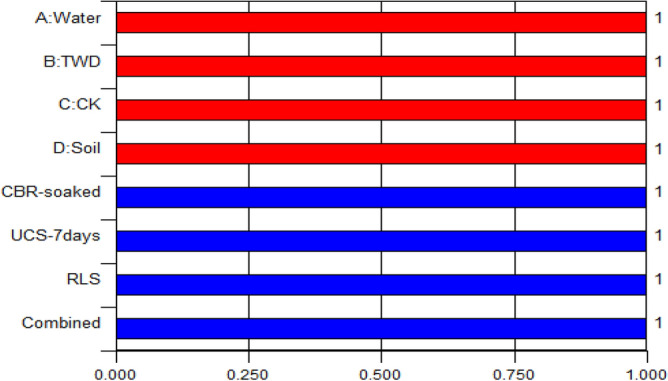
Bar Graph for the desirability function.

The analytical outputs derived from the 4-components mixture design optimization are presented graphically through ramps plot and bar charts for clarity and understanding of the obtained results. The ramp and bar chart illustrates the optimal solution for the factor levels in red colours while the target responses are in blue colouration to elucidate the obtained desirability function computation outputs as shown in Figs. 18–19. The graphical optimization results presented indicated the explanatory and response variables’ desirability score of 1.0, while the combined optimal desirability score of 1.0 was calculated which signifies adequate performance taking into consideration the imposed factors criteria.

#### Optimization trace plot

**Figure 20:**
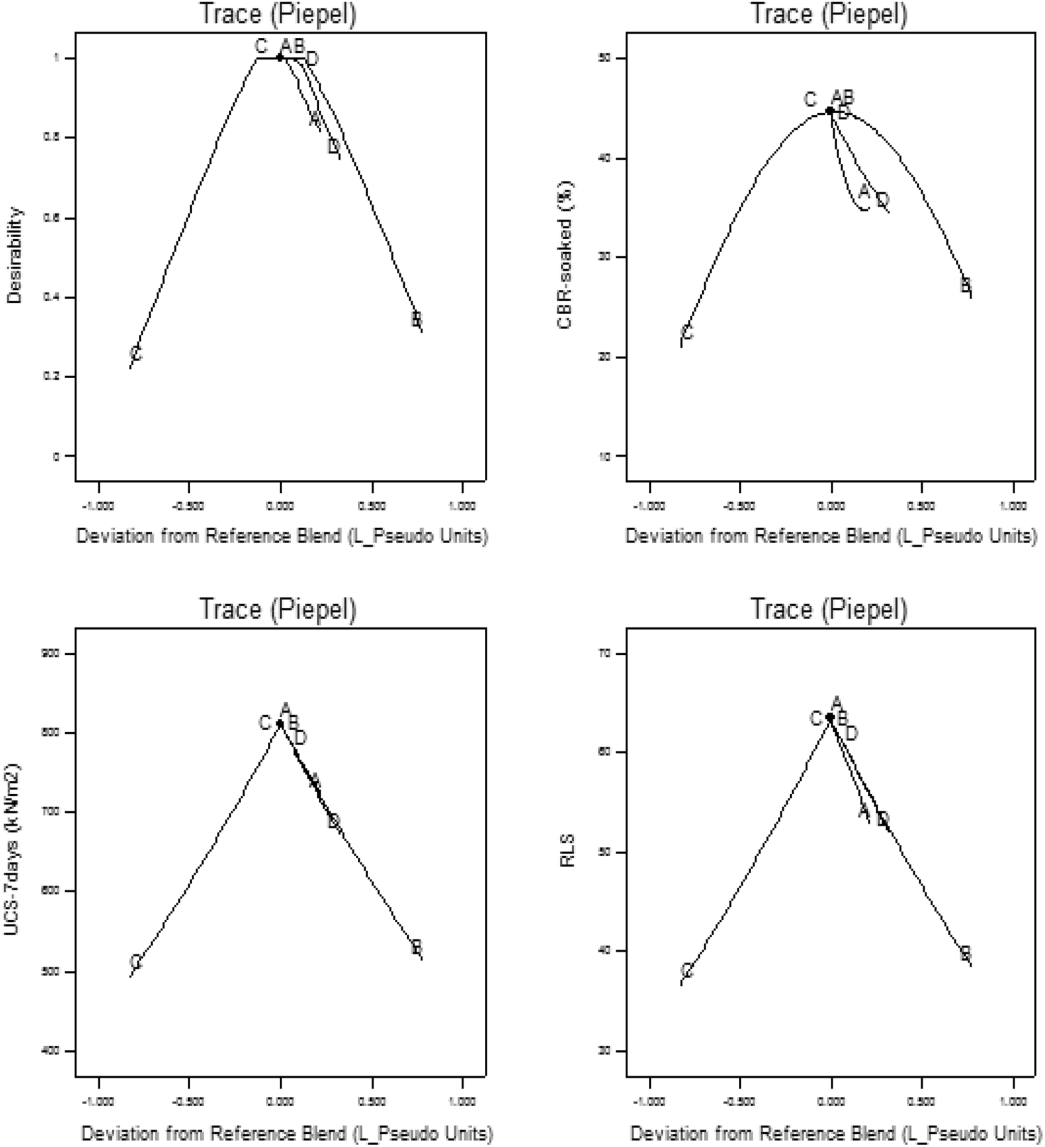
Optimization trace piepel plot.

Trace plots are deployed to assess all mixture components’ effects in the feasible experimental space considering the derived optimal responses and it’s similar to perturbation plots adapted for analysis in non-mixture designs. The referenced optimized mixture blend is derived using this graphical assessment tool which is aimed at obtaining the target response sensitivity when related to the formulation deviation near the reference blend. The trace plot adapted for this evaluation study is through the Piepel direction along which the fractions of varying amounts of all the other mixture components are held constant. The plot presents the pseudo-coded units ranging from -1 to + 1 on the x-axis to indicate significant locations relative to the coded scale as shown in Fig. 20. The results in the plot indicate that components C and B were positioned between − 1.0 and − 0.5 and 0.5–1.0 respectively on the coded units’ axis.

#### Optimization contour plot

**Figure 21:**
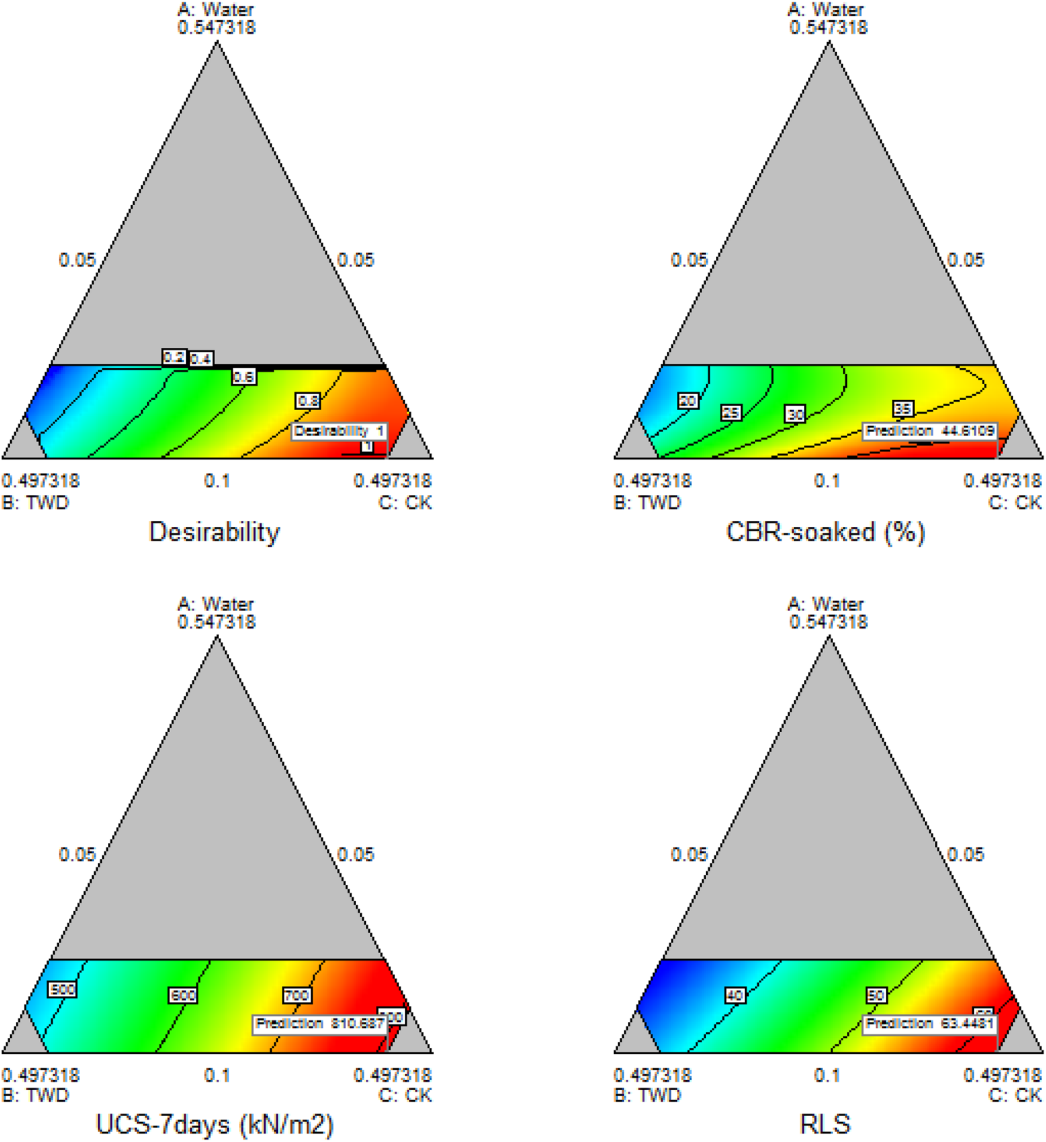
Contour plots for the optimal solution.

The contour plot is a two-dimensional graphical analysis computational tool that helps the constrained experimental space visualization to observe the numerical factor points against the mixture components within the simplex through an iterative optimization solution as shown in Fig. 21. The presented graphical plots indicated a three-edged triangular plot with three of the four mixture components simplex showing the optimized ratio of 0.54718, 0.497318 and 0.497318 for water, TWD and CK respectively.

#### 3D surface plots

**Figure 22:**
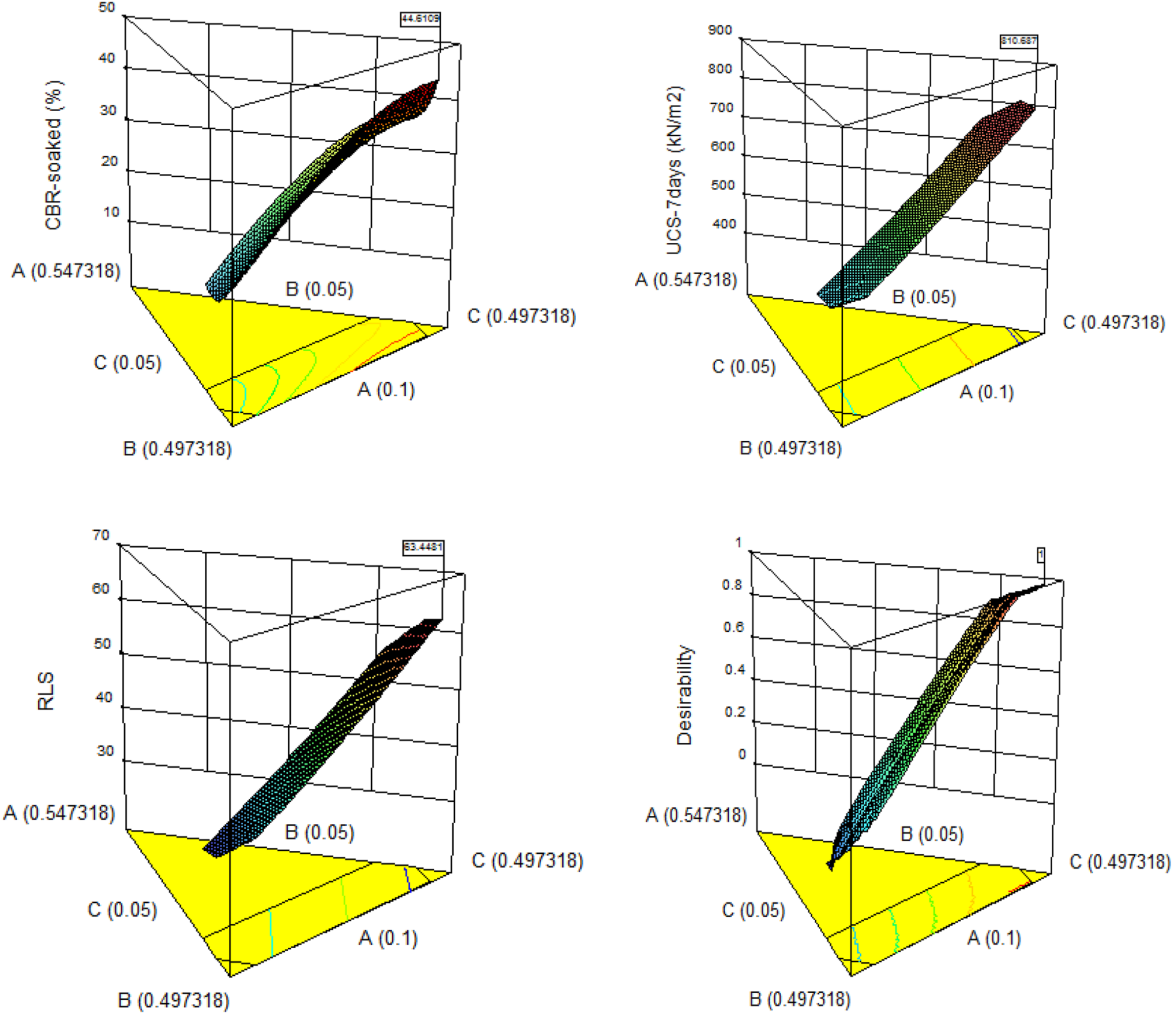
3D plot for the optimal solution.

3-dimensional surface plots are graphical illustrations of the interactions and connectivity behaviour between the factor levels to the response variables. The 3-dimensional graphs for the mixture components concerning the dependent variables and desirability function optimal solution and presenting the corresponding experimental design points’ response surface is shown in Fig. 22. The plotted results indicated optimal responses of 44.611%, 810.687 kN/m^2^ and 63.448% for CBR, UCS and RLS respectively.

### EVD-Model post-analysis and simulation

Post-analysis and model simulation was carried out after the EVD-model development, numerical and graphical optimization to validate the derived factor values, point prediction and confirmation to achieve sample means and coefficient table. Also, a simulation exercise of the EVD-model generated to assess its applicability and performance is further achieved.

#### Point prediction computation

This post-analysis tool essentially provides an avenue for the factor values to be reviewed which utilizes the fit statistical computation of the developed model and the prescribed settings for the factor tools to determine interval estimates after the analysis is successfully carried out as presented in Table 40. The tabulated outputs showed standard deviation of 1.877, 51.757 and 2.999, low to high confidence interval (CI) values of 39.553–50.506, 734.737–881.168 and 58.893–67.381 for CBR, UCS and RLS response parameters respectively.

**Table 40:** Point prediction.

Response	Predicted mean	Predicted median	Std. dev	CI for 95% CI low	Mean 95% CI high	99% of 95% TI low	Population 95% TI high
CBR-soaked	44.8624	44.8427	1.87707	39.5531	50.5061	31.2842	60.8356
UCS-7 days	806.289	805.458	51.7569	734.737	881.168	561.075	1093.9
RLS	63.0658	63.0302	2.99969	58.8934	67.3811	48.5336	79.4184

#### Coefficient-table

The coefficient table presents the magnitude combination ratio of the factor levels which evaluates the 4-components TWD-CK-soil mixture blend aimed at improving the engineering behaviour of expansive soil for pavement construction as shown in Table 41. From the presented multi-criteria optimization analysis, quadratic was preferred for CBR response and linear models for both UCS and RLS responses to obtain a generalization of experimental data. The legend column was used to assess the p-values for every term of the models where *p*-values < 0.01 signifies the best measures for strong significance presented in red, *p*-values ≤ 0.05 and ≥ 0.01 indicates a moderate level of significance presented in green, *p*-values ≤ 0.10 and ≥ 0.05 signifies a slight level of significance presented in blue while *p*-values ≥ 0.10 indicate a level of non-insignificant displayed in black.

**Table 41:** Coefficient table.

Response	A	B	C	D	AB	AC	AD	BC	BD	CD
Sqrt(CBR-soaked)	21.3623	4.58485	6.65234	5.11559	− 24.896	− 22.783	− 18.253	2.76667	− 6.4705	− 0.5015
p =	< 0.0001	< 0.0001	< 0.0001	< 0.0001	0.0229	0.0321	0.0658	0.0053	0.1109	0.8892
Sqrt(UCS-7 days)	21.3183	21.8101	29.2966	20.672						
p =	< 0.0001	< 0.0001	< 0.0001	< 0.0001						
Sqrt(RLS)	4.80229	5.9269	8.21968	5.63527						
*p* =	< 0.0001	< 0.0001	< 0.0001	< 0.0001						
Legend	*p* < 0.01	0.01 ≤ p < 0.05	0.05 ≤ *p* < 0.10	*p* ≥ 0.10						

#### EVD model simulation

**Figure 23:**
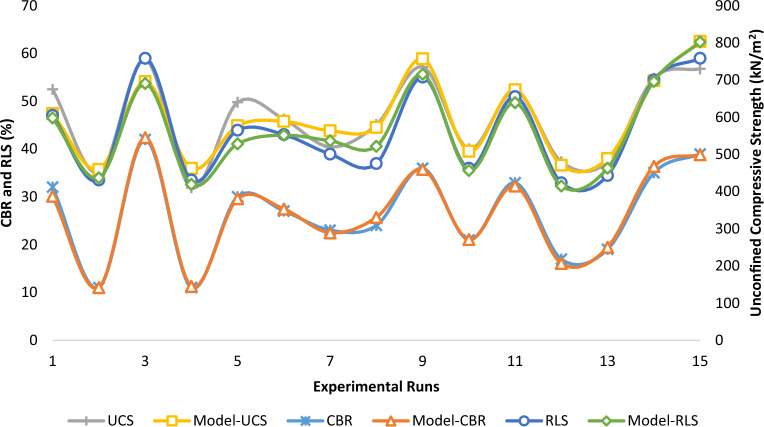
Actual versus EVD-model predicted responses.

The simulation of the EVD-model is carried out after the derivation of the coefficient table and post-analysis process. It is also the last phase of the statistical analysis of the 4-components mixture design optimization and is executed to validate the necessary statistical assessments carried out to develop the EVD-model to simulate real-life conditions which provide adequate guidance for contractors, field operators, designers and consultants on the applicability and performance of the developed EVD-model. The simulation procedure involves comparing statistically the experimental or actual values with the simulated model results as shown in Fig. 23 using analysis of variance (ANOVA) at a 95% confidence interval. The statistical evaluation of the simulated vs actual results was achieved using Microsoft excel software and the outputs are presented in Tables 42, 43, 44. From the computed results, P(F <  = f) two-tail of 0.9965, 0.9888 and 0.9997 was computed which is greater than 0.05 for CBR, UCS and RLS target responses respectively. The derived statistical results indicate that there is no significant difference between the laboratory or actual and model predicted results which implies acceptable model estimation accuracy in line with the research findings of Aju et al.^71^.

**Table 42:** t-test Two-sample assuming equal variances for CBR response.

	CBR	Model-CBR
Mean	26.672	26.656587
Variance	94.079	94.211896
Observations	15	15
Pooled Variance	94.145	
df	28	
t Stat	0.0044	
P(T < = t) one-tail	0.4983	
t Critical one-tail	1.7011	
P(T < = t) two-tail	0.9965	
t Critical two-tail	2.0484

**Table 43:** t-Test two-sample assuming equal variances for UCS response.

	UCS	Model-UCS
Mean	595.9552	595.3734
Variance	13,111.67	12,109.96
Observations	15	15
Pooled Variance	12,610.82	
df	28	
t Stat	0.014187	
P(T < = t) one-tail	0.494391	
t Critical one-tail	1.701131	
P(T < = t) two-tail	0.988782	
t Critical two-tail	2.048407

**Table 44:** t-Test two-sample assuming equal variances for RLS response.

	*RLS*	*Model-RLS*
Mean	43.9248	43.92341
Variance	93.67385	88.69949
Observations	15	15
Pooled Variance	91.18667	
df	28	
t Stat	0.0004	
P(T < = t) one-tail	0.499842	
t Critical one-tail	1.701131	
P(T < = t) two-tail	0.999684	
t Critical two-tail	2.048407	

## Comparison with previous studies

The recent hotspot in the field of materials and construction engineering is the utilization of waste residue in conjunction with a suitable optimization technique in soil amelioration protocols. The use of waste residue is a result of the high cost of cement, depletion of the ozone layer due to cement production and as well as promoting sustainable environment whereas the deployment of optimization technique is to aid in model building and as well reduce the process of the random number of experiments experienced in the trial and error approach. Thus, this necessitated the deployment of extreme vertex design of experiment approach in the current study. Notably, in this current experimental study, the CBR upshots of soil-calcined kaolin mixtures with tile waste dust demonstrated an appreciating trend with the addition of additive dosages. In contrast to the study of Alaneme et al.^9^ who engaged EVD in the optimization of a single additive for the amelioration of expansive soil, they reported an increasing CBR with the incorporation of additive material. However, both studies used black expansive clay soils but in their study, a single additive was considered whereas in this current study binary additives were considered and this might not be unrelated to the rate of strength gained. Ikeagwuani et al.^59^ established coherently that optimization of multi-additives via means of Taguchi mixture experimental design in a certain way influenced the mechanical response (differential free swell, California bearing ratio, and unconfined compressive strength) of expansive soil. Surprisingly, the durability response recorded in this study increased with an increase in the dose of additives to a peak value of 59%. The durability outcome for the optimal soil additive mixture fell short of the 80% requirement and it seems to be consistent with those of^66^. In summary, the extreme vertex design optimization strategy used in this experimental study endorses the explorations of^9,44^ who disclosed the effectiveness and efficiency of EVD in civil engineering materials.

## Conclusion

This study documents the upshots of successful soil re-engineering protocols of a weak soil ameliorated with the combination of TWD and CK based on the principles of EVD strategy. Based on AASHTO classification and USCS scheme, the soil in its original form falls under the class of A-7–6 material with group index of 14 and CH, respectively. The upshot of this optimization study indicates that using the mixture ratio of 0.10:0.23:0.32:0.35 for water, TWD, CK and soil respectively, optimally enhanced the key parameters including CBR, UCS and RLS. The documented rate of enhancement in the soil’s mechanical might not be far from the pozzzolanic interplay within the soil structure as a result of the introduction of additive materials, which was later confirmed qualitatively via microstructural experiments such as scanning electron microscopy (SEM). From the geo-engineering point of view, the benefit of the present investigation entails in numerous folds: [i] the integration of waste materials as ameliorants promotes a sustainable environment thereby regulating the depletion of natural resources and possible occurrence of global warming, [ii] it will create a cost-effective strategy in the eradication of mammoth amount of waste materials are generated into the society thereby reducing nuisance and cleaner environment and [iii] in the course of infrastructural developments, the practice of waste materials reutilization in soil re-engineering as either cement or lime surrogate materials facilitates lessening in cost of Civil engineering projects. The use of EVD strategy have shown a huge success thus, the use of other optimization techniques such as Taguchi, response surface methodology etc. would be a welcome development as it will create room for further studies and as much a more efficient optimal ratio could be achievable.

## Data Availability

All data generated or analyzed during this study are included in this published article.

## References

[1] Ikeagwuani CC, Nwonu DC (2019). Emerging trends in expansive soil stabilisation. A review. J. Rock Mech. Geotech. Eng..

[2] Soltani A, Deng A, Taheri A (2018). Swell-compression characteristics of a fiber-reinforced expansive soil. Geotext. Geomembr..

[3] Nalbantoglu Z, Al-Rawas AA, Goosen MFA (2006). Lime stabilisation of expansive clay”. Expansive soils: Recent advances in characterisation and treatment.

[4] Petry TM, Little DN (2002). Review of stabilization of clays and expansive soils in pavements and lightly loaded structures-history, practice and future. J. Mater. Civ. Eng..

[5] Alaneme GU, Mbadike ME, Attah IC, Udousoro IM (2022). Mechanical behavior optimization of sawdust ash and quarry dust concrete using adaptive neuro-fuzzy inference system. Innov. Infrastruct. Solut..

[6] Attah IC, Etim RK, Usanga IN (2021). Potentials of cement kiln dust and rice husk ash blend on strength of tropical soil for sustainable road construction material. IOP Conf. Ser. Mater. Sci. Eng..

[7] Attah IC, Okafor FO, Ugwu OO (2022). Durability performance of expansive soil ameliorated with binary blend of additives for infrastructure delivery. Innov. Infrastruct. Solut..

[8] Attah IC, Etim RK, Ekpo DU, Usanga IN (2022). Effectiveness of cement kiln dust-silicate based mixtures on plasticity and compaction performance of an expansive soil. Építőanyag J. Silicate Based and Composite Materials..

[9] Alaneme GU, Attah IC, Etim RK, Dimonyeka MU (2022). Mechanical properties optimization of soil - cement kiln dust mixture using extreme vertex design. Int. J. Pavement Res. Technol..

[10] Etim RK, Attah IC, Eberemu AO, Yohanna P (2019). Compaction behaviour of periwinkle shell ash treated lateritic soil for use as road sub-base construction material. J. Geoeng..

[11] Etim RK, Attah IC, Yohanna P (2020). Experimental study on potential of oyster shell ash in structural strength improvement of lateritic soil for road construction”. Int. J. Pavement Res. Technol..

[12] Attah IC, Etim RK, Yohanna P, Usanga IN (2021). Understanding the effect of compaction energies on the strength indices and durability of oyster shell ash-lateritic soil mixtures for use in road works. Eng. Appl. Sci. Res..

[13] Onyelowe KC, Alaneme GU, Igboayaka C, Orji F, Ugwuanyi H, Van Bui D, Manh NV (2019). Schefe optimization of swelling, California bearing ratio, compressive strength and durability potentials of quarry dust stabilized soft clay soil. Mater. Sci. Energy Technol..

[14] Jalal FE, Mulk S, Memon SA, Jamhiri B, Naseem A (2021). Strength, hydraulic and microstructural characteristics of expansive soils incorporating marble dust and rice husk ash. Adv. Civil Eng..

[15] Oluremi JR, Adedokun SI, Osuolale OM (2012). Stabilization of poor lateritic soils with coconut husk ash. Int. J. Eng. Res. Tech..

[16] Jimoh YA, Apampa OA (2014). An evaluation of the influence of corn cob ash on the strength parameters of lateritic soils. Civil Environ. Res..

[17] Osinubi KJ, Akinmade OB, Eberemu AO (2009). Stabilization potential of locust bean waste ash on black cotton soil. J. Eng. Res..

[18] Osinubi KJ, Eberemu AO, Akinmade OB (2016). Evaluation of strength characteristics of tropical black clay treated with locust bean waste ash. Geotech. Geol. Eng..

[19] Sujatha ER, Dharini K, Bharathi V (2015). Influence of groundnut shell ash on strength and durability properties of clay Geomechan. Geoeng..

[20] Moses G, Etim RK, Sani JE, Nwude M (2019). Desiccation-induced volumetric shrinkage characteristics of highly expansive tropical black clay treated with groundnut shell ash for barrier consideration. Civil Environ. Res..

[21] Sani JE, Yohanna P, Etim RK, Attah IC, Bayang F (2019). Unconfined compressive strength of compacted lateritic soil treated with selected admixtures for geotechnical applications. Niger. Res. J. Eng. Environ. Sci..

[22] Alaneme GU, Onyelowe KC, Onyia ME, Van Bui D, Mbadike EM, Dimonyeka MU, Attah IC, Ogbonna C, Iro UI, Kumari S, Firoozi AA, Oyagbola I (2020). Modelling of the swelling potential of soil treated with quicklime-activated rice husk ash using fuzzy logic. Umudike J. Eng. Technol..

[23] Alaneme GU, Elvis MM (2021). Experimental investigation of Bambara nut shell ash in the production of concrete and mortar. Innov. Infrastruct. Solut..

[24] Ramonu JAL, Ilevbaoe JO, Ayandalfedapo S, Modupe AE, Adeniyi OM, Adewole TA (2018). Geotechnical properties of lateritic soil stabilized with yam peel ash for subgrade construction. In. J. Civil Eng. Technol..

[25] Attah IC, Agunwamba JC, Etim RK, Ogarekpe NM (2019). Modelling and predicting of CBR values of lateritic soil treated with metakaolin for road material. ARPN J. Eng. Appl. Sci..

[26] Qiu Y, Sego DC (2001). Laboratory properties of mine tailings. Can. Geotech. J.

[27] Onyelowe KC, Duc BV (2018). Predicting subgrade stiffness of nanostructured palm bunch ash stabilized lateritic soil for Transport geotechnics purposes. J. GeoEng..

[28] Ekpo DU, Fajobi AB, Ayodele AL (2020). Response of two lateritic soils to cement kiln dust—periwinkle shell ash blends as road sub-base materials. Int. J. Pavement Res. Technol..

[29] Ekpo DU, Fajobi AB, Ayodele AL, Etim RK (2021). Potentials of cement kiln dust-periwinkle shell ash blends on plasticity behaviour of two tropical soils for use as sustainable construction materials”. IOP Conf. Series: Mater. Sci. Eng..

[30] Adetayo OA, Umego OM, Faluyi F, Odetoye AO, Bucknor AO, Busari AA, Sanni A (2021). Evaluation of pulverized cow bone ash and waste glass powder on the geotechnical properties of tropical laterite. SILICON.

[31] Ikeagwuani CC, Obeta IN, Agunwamba JC (2019). stabilization of black cotton soil subgrade using sawdust ash and lime. Soils Found..

[32] Sani JE, Yohanna P, Chukwujama IA (2020). Effect of rice husk ash admixed with treated sisal fibre on properties of lateritic soil as a road construction material. J. King Saud. Univ. Eng. Sci..

[33] Gedefaw A, Yifru BW, Endale SA, Habtegebreal BT, Yehualaw MD (2022). Experimental investigation on the effects of coffee husk ash as partial replacement of cement on concrete properties. Adv. Mater. Sci. Eng..

[34] Chen W, Zhang G, Tao Q, Yu L, Li T, Guan X (2022). Experimental research on mix ratio of construction waste cemented filling material based on response surface methodology. Adv. Mater. Sci. Eng..

[35] Butt WA, Gupta K, Jha JN (2016). Strength behaviour of clayey soil stabilized with sawdust ash. Geoengineering.

[36] Buazar F (2019). Impact of biocompactible nanosilica on green stabilization of subgrade soil. Sci. Rep..

[37] Al-Amoudi OSB, Al-Homidy AA, Maslehuddin M, Saleh TA (2017). Method and mechanisms of soil stabilization using electric arc furnace dust. Sci. Rep..

[38] Osinubi KJ, Yohanna P, Eberemu AO (2015). Cement modification of tropical black clay using iron ore tailing as admixture. J. Transp. Geotech..

[39] Jalal FE, Xu Y, Jamhiri B (2020). Memon SA (2020) On the recent trends in expansive soil stabilization using calcium-based stabilizer materials (CSMs): A comprehensive review. Adv. Mater. Sci. Eng..

[40] Etim RK, Ekpo DU, Etim GU, Attah IC (2021). Evaluation of lateritic soil stabilized with lime and periwinkle shell ash (PSA) admixture bound for sustainable road materials. Innovative Infrastructure Solutions.

[41] Gholampour AA, Gandomi AH, Ozbakkaloghu T (2017). New formulations for mechanics properties of recycled aggregate concrete using gene expression programming. Constr. Build. Mater..

[42] Onyia ME, Ikeagwuani CC, Egbo MC (2022). Effect of pressed palm oil fruit fibre on mechanical properties of sandcrete blocks using Taguchi-grey relational analysis. Clean. Mater..

[43] Mohammed M, Sharafati A, Al-Ansari N, Yaseen M (2020). Shallow foundation settlement quantification: Application of hybridized adaptive neuro fuzzy inference system model. Adv. Civil Eng.

[44] Alaneme GU, Iro UI, Milad A (2023). Mechanical properties optimization and simulation of soil-saw dust ash blend using extreme vertex design (EVD) method. Int. J. Pavement Res. Technol..

[45] Sridhar J, Shinde GB, Vivek D, Naseem K, Gaur P, Patil P, Tesema MT (2022). Response surface methodology approach to predict the flexural moment of ferrocement composites with weld mesh and steel slag as partial replacement for fine aggregate”. Adv. Mater..

[46] Onyelowe KC, Iqbal M, Jalal FE, Onyia ME, Onuoha IC (2021). Application of 3-algorithm ANN programming to predict the strength performance of hydrated-lime activated rice husk ash treated soil. Multiscale Multidiscip. Model. Exp. Design.

[47] Khan K, Jalal FE, Iqbal M, Khan MI, Amin MN, Al-Faiad MA (2022). Predictive modelling of compression strength of waste PET/SCM blended cementitious grout using gene expression programming”. Materials.

[48] Onyelowe KC, Jalal FE, Onyia ME, Onuoha IC, Alaneme GU (2021). Application of gene expression programming to evaluate strength characteristics of hydrated-lime-activated rice husk ash-treated expansive soil. Appl. Comput. Intell. Soft Comput..

[49] Yildizel SA, Tayeh BA, Calis G (2020). Experimental and modelling study of mixture design optimisation of glass fibre-reinforced concrete with combined utilisation of Taguchi and extreme vertices design techniques. J. Mater. Res. Technol..

[50] Nwonu DC, Onyia ME (2023). Novel grey-vector optimization of desiccation induced shrinkage and strength of industrial based soil-composite binder as sustainable construction material. J. Mater. Cycles Waste Manag..

[51] Khan K, Salami BA, Iqbal M, Amin MN, Ahmed F, Jalal FE (2022). Compressive strength estimation of fly ash / slag based green concrete by deploying artificial intelligence models. Materials.

[52] Onyelowe KC, Alaneme GU, Van Bui D, Van Nguyen M, Ezugwu C, Amhadi T, Sosa F, Orji F, Ugorji B (2019). Generalized review on EVD and constraints simplex method of materials properties optimization for civil engineering. Civil Eng. J..

[53] Federal Highway Department. General specifications (Roads and Bridges). Nigeria: Federal ministry of works and housing; 1997.

[54] British Standard (BS) 1377 (1990b) Methods of testing soils for civil engineering purposes, part 4.

[55] Ola SA (1974). Need for estimating cement requirement for stabilization of lateritic soils. J. Transp. Eng. Div..

[56] Jaritngam O, Somchaimeek P, Taneeranon P (2014). Feasibility of lateritic-cement mixture as pavement base course aggregate. Iran. J. Sci. Technol. Trans. Civil Eng..

[57] Ikeagwuani CC, Agunwamba JC, Nwankwo CM, Eneh M (2020). Additives optimization for expansive soil subgrade modification based on Taguchi grey relational analysis. Int. J. Pavement Res. Technol..

[58] Ikeagwuani CC, Nwonu DC, Onah HN (2020). Min-max fuzzy goal programming - Taguchi model for multiple additives optimization in expansive soil improvement. Int. J. Numer. Anal. Methods Geomech..

[59] Attah IC, Okafor FO, Ugwu OO (2021). Optimization of California bearing ratio of tropical black clay soil treated with cement kiln dust and metakaolin blend. Int. J. Pavement Res. Technol..

[60] Ezeokpube GC, Ahaneku IE, Alaneme GU, Attah IC, Etim RK, Olaiya BC, Udousoro IM (2022). Assessment of mechanical properties of soil-lime-crude oil contaminated soil blend using regression model for sustainable pavement foundation construction. Adv. Mater. Sci. Eng..

[61] Ezeokpube GC, Alaneme GU, Attah IC, Udousoro IM, Nwogbo D (2022). Experimental investigation of crude oil contaminated soil for sustainable concrete production architecture. Struct. Constr..

[62] Attah IC, Okafor FO, Ugwu OO (2021). Experimental and optimization study of unconfined compressive strength of ameliorated tropical black clay. Eng. Appl. Sci. Res..

[63] Wilson MJ (1987). A handbook of determinative methods in clay mineralogy.

[64] Olokode OS, Aiyedun PO, Kuye SI, Adekunle NO, Lee WE (2010). Evaluation of a clay mineral deposit in Abeokuta, South-West, Nigeria. J. Nat. Sci. Eng. Technol..

[65] Nwonu DC, Ikeagwuani CC (2019). Evaluating the effect of agro-based admixture on lime-treated expansive soil for subgrade material. Int. J. Pavement Eng..

[66] Sani JE, Etim RK, Joseph A (2018). Compaction behaviour of lateritic soil-calcium chloride mixtures. Geotech. Geol. Eng..

[67] Etim RK, Attah IC, Ekpo DU, Usanga IN (2021). Evaluation on stabilization role of lime and cement in expansive black clay-oyster shell ash composite. Transpor. Infrastruct. Geotechnol..

[68] Al-Rawas A, McGown A (1999). Microstructure of Omani expansive soils. Can. Geotech. J..

[69] Al-Rawas A (2002). Microfabric and mineralogical studies on the stabilization of an expansive soil using cement by-pass dust and some types of slag. Can. Geotech. J..

[70] Mirzababaei M, Yasrobi S, Al-Rawas A (2009). Effect of polymers on swelling potentials of expansive clays. Proc. Inst. Civ. Eng. Ground Improv..

[71] Aju DE, Onyelowe KC, Alaneme GU (2021). Constrained vertex optimization and simulation of the unconfined compressive strength of geotextile reinforced soil for flexible pavement foundation construction. Clean. Eng. Technol..

